# Identification of *Malus sieversii* ABA receptor PYL8 interacting proteome using Y2H-seq

**DOI:** 10.48130/forres-0025-0012

**Published:** 2025-06-30

**Authors:** Zhen Fang, Meiyun Li, Jing Li, Juan Ma, Kai Zhang, Chunyu Yang, Chunxiu Ye

**Affiliations:** College of Forestry and Landscape Architecture, Xinjiang Agricultural University, Urumqi 830052, PR China

**Keywords:** *Malus sieversii*, ABA, Receptors, PYL8, Interacting, Proteome

## Abstract

The seed dormancy of *Malus sieversii* belongs to the comprehensive type of dormancy, abscisic acid (ABA) is one of the important hormones to lift the seed dormancy. The ABA receptor pyrabactin resistance-like (PYL) protein is involved in seed germination and various stress responses. Fourteen *MsPYL* genes were evaluated in the genome of *Malus sieversii*. Phylogenetic analyses demonstrated that MsPYL proteins can be divided into three groups. The promoter regions of *MsPYL* genes contain cis-acting elements associated with expression induction by abiotic stresses such as abscisic acid, drought, and low temperature. *Arabidopsis* transgenic lines overexpressing *MsPYL8* exhibited a markedly reduced germination rate, an extended growth period, and a reduction in the biomass of the aboveground parts compared to the wild type. Additionally, we found that MsPYL8 interacted with 30 proteins, including low-temperature-induced 65 kDa protein-like (LTI) and late embryogenesis abundant (LEA). The results demonstrated that PYL8 binds to ABA during seed germination, inhibits the dephosphorylation activity of protein phosphatases type 2C (PP2C), and activates the transmission of sucrose non-fermenting-1-related protein kinases 2s (SnRK2s) to abscisic acid insensitive 5 (ABI5) signaling, thereby activating the expression of ABA-responsive genes to regulate ABA signal transduction. The screening and validation of PYL8 interaction with LTI, and LTI interaction with ABI5, provides a foundation for further elucidation of the mechanism of *PYL* gene involvement in *Malus sieversii* seed dormancy lifting under the regulation of the ABA signaling pathway.

## Introduction

*Malus sieversii* is one of the main ancestors of cultivated apples, known for its rich genetic diversity and disease-resistant traits^[1]^. However, its population renewal is hindered by deep seed dormancy, necessitating the use of artificial near-natural breeding methods to support conservation efforts^[2]^. Exploring efficient germination methods and elucidating the mechanisms underlying seed dormancy release in *Malus sieversii* are critical for the restoration and preservation of wild apple populations. Seed germination is a vital process for the reproduction of flowering plants. ABA regulates multiple stages of plant development, including seed dormancy regulation, inhibition of embryo-to-seedling transition, and early seedling growth^[3,4]^. Seed dormancy and germination are modulated by the interplay between ABA and gibberellin (GA) biosynthesis and signaling transduction pathways^[5,6]^. In mature seeds, endogenous ABA accumulates to establish and sustain dormancy. During water-absorbent expansion (WAE), ABA levels initially decline but rise again in later stages, suggesting a dynamic role in dormancy control^[7]^. During seed germination initiation, endogenous ABA levels decrease, while GA content increases in response to imbibition and stratification treatments^[8]^. ABA signaling operates through three key phases: (1) ABA production and transport, (2) ABA signal perception and transmission, and (3) ABA-responsive and regulation. The PYR/PYL/RCAR family of ABA receptors serves as the critical entry point for ABA signal perception^[9,10]^. ABA-dependent gene expression is primarily regulated by ABA-responsive element-binding proteins (AREBs), and ABRE binding factors (ABFs). The core ABA signaling pathway consists of three major components: ABA receptors (PYR/PYL/RCAR), protein phosphatase 2C (PP2C), and sucrose non-fermenting-1-related protein kinases 2 (SnRK2) family members^[11−13]^. Under normal conditions, PP2C acts as a negative regulator by binding to and dephosphorylates SnRK2, thereby maintaining SnRK2 in an inactive state and blocking ABA signal transmission. When plants experience stress, ABA rapidly accumulates and binds to PYL receptors. This PYL-ABA complex then interacts with PP2C, inhibiting PP2C's dephosphorylation activity^[14]^. Consequently, PP2C-mediated suppression of SnRK2 is relieved, allowing SnRK2 phosphorylation of downstream targets, including AREB/ABF transcription factors lifting, which subsequently induce expression of ABA-responsive genes to mediate stress adaptation^[15−19]^.

Studies on the 14 *PYL* genes in *Arabidopsis thaliana* demonstrated that 11 *PYLs* exhibit significant expression changes during seed germination under exogenous ABA regulation. Notably, *PYL11* and *PYL12* showed seed-specific expression in mature seeds and played a positive regulatory role in ABA-mediated germination^[20]^. Genetic screens for ABA-insensitive germination identified key mutants abscisic acid insensitive 3 (*ABI3*), *ABI4*, and *ABI5*. Further characterization demonstrated that *abi4* and *abi5* mutants display altered ABA sensitivity in seed germination, dormancy maintenance, and stomatal regulation, with these genes showing seed-specific expression during early germination stages^[21,22]^. While the biochemical functions of PYR/PYL/RCAR receptors have been well established^[11]^, several aspects remain incompletely understood, including their subcellular localization patterns, post-translational modifications, and protein turnover regulation. Recent findings identified DDB1-ASSOCIATED1 (DDA1) as a key regulator of ABA signal desensitization through ubiquitin-mediated degradation of PYL8, PYL4, and PYL9^[23]^. Additionally, core signaling components are regulated through phosphorylation cascades involving reciprocal modulation between ABA receptors, SnRK2 kinases, and the target of rapamycin (TOR) kinase. For instance, ABA receptors can be inactivated through phosphorylation by early flowering 1 (EL1)-like casein kinase or targeted for degradation via TOR-mediated pathways^[24,25]^. Most of studies are limited to illustrating the effects of biochemical processes and protein translational modifications.

Emerging evidence demonstrates the functional specialization among PYL family members. PYL5, which localizes to both the cytoplasm and nucleus, activates ABA signaling through direct inhibition of clade A PP2Cs. The analysis of PYL5 interactions, employing covalent chemical capture coupled with HPLC-MS/MS, revealed its associations with three key proteins: the ubiquitin receptor RAD23C, the COP9 signalosome subunit CSN1, and cyclase-associated protein CAP1^[26]^. Notably, PYL8 exhibits ABA-independent functions by interacting with MYB transcription factors (MYB77, MYB44, and MYB73) to enhance auxin signaling and promote lateral root growth, operating outside the canonical PYL-PP2C-SnRK2 cascade^[27]^. The biochemical characterization of PYL13 indicates its unique capacity to inhibit clade A PP2Cs in an ABA-independent manner, while PYL10 may participate in heteromeric coordination^[28]^. Furthermore, PYL members show distinct physiological roles: PYL2 specifically regulates ABA-mediated stomatal closure, whereas PYL4 and PYL5 are essential for the CO_2_ response in guard cells^[29]^. At the transcriptional level, ABA signaling components are tightly regulated. Typically, repressor proteins modulate PP2C activity, as exemplified by enhancers of ABA co-receptor1 (EAR1) mutants. These mutants act as negative regulators by interacting with downstream PP2Cs and enhancing their activity, thereby attenuating ABA signaling^[30]^.

Most proteins regulate biological processes by recruiting binding partners to form functional complexes^[31]^. The yeast two-hybrid (Y2H) assay is one of the most common methodologies to search for protein interactions, and its large-scale application has been constrained by cost and labour resources of repetitive screening. In 2017, researchers developed CrY2H-seq, an enhanced Y2H-based inter-library screening technology coupled with next-generation sequencing. This method enables large-scale interaction screening without limitations imposed by bait protein numbers. However, CrY2H-seq has several drawbacks, including the high cost of constructing interacting libraries, interference from self-activated proteins, and the short read lengths characteristic of second-generation sequencing^[32]^. Subsequent advancements in 2021, led to the development of Y2H-seq, which combines the sensitivity of Y2H with high-throughput sequencing. This optimized system reduced total experiment time by 66% and the cost by 89%^[31]^. More recently, further improvements to CrY2H-seq have been achieved through enhanced detection of self-activated proteins. By third-generation sequencing technologies (micro and nanopore sequencing) with systematic bait library screening, researchers significantly reduced false-positive rates While improving the detection efficiency of true interaction pairs^[33]^. These technological advances have made high-throughput sequencing combined with Y2H screening a robust and feasible approach for large-scale protein interaction studies.

The *PYL* gene family members in *Malus sieversii* were identified through bioinformatics methods analyzed their phylogenetics, gene structure, conserved structural domains, cis-acting elements, and expression patterns. Using Y2H-seq screening followed by yeast point-to-point verification and bimolecular fluorescence complementation (BiFC) assays, we identified and validated MsPYL8 interacting proteome. In addition, transgenic *Arabidopsis* lines overexpressing *MsPYL8* were generated, confirming its functional role in ABA-mediated seed germination. These results will provide a scientific basis for further research into the molecular mechanism of ABA signal transduction in regulating seed germination.

## Materials and methods

### Plant materials and treatments

Seeds of *Malus sieversii* were collected in October 2023 from the wild fruit forests in Emin County, Tacheng, Xinjiang, China (80°47' E−83°58' E, 43°20' N−46°21' N). The control group (CK0) were seed samples stored at room temperature for 0 d. The treatment group (M0) were CK0 seeds soaked in water for 24 h, followed by low-temperature sand stratification at 4 °C for four durations (30, 60, 90, and 120 d), designated as M30, M60, M90, and M120, respectively. For each period, three biological replicates were prepared. All seed samples were flash-frozen in liquid nitrogen and later stored at −80 °C.

### Identification and characterization of *PYL* gene in *Malus sieversii*

Hidden Markov model files (PF10604) of the conserved structural domains of PYL were downloaded from the Pfam^[34]^ database (http://pfam.xfam.org/), and members of the PYL family of *Malus sieversii* were screened using HMMER. *Malus sieversii* and *Malus domestica* genomic data were downloaded from the Apple Database^[35]^ (http://bioinfo.bti.cornell.edu/apple_genome/). Proteins were searched using Hmmer Search of the HMMER software, setting the e value to 1 × 10^−20^. For further confirmation of all candidate genes, all were searched by Pfam, CDD^[36]^, (www.ncbi.nlm.nih.gov/cdd), and SMART^[37]^ (http://smart.embl-heidelberg.de/) databases for conserved structural domain analysis, identified with PYR_PYL_RCAR_like structural domain (cd07821). The duplicates were de-duplicated to finally obtain the members of the PYL family of *Malus sieversii*. The above methods were used to obtain *PYL* gene family members and protein sequences of *Arabidopsis thaliana*, *Populus trichocarpa*, rice (*Oryza sativa*), wheat (*Triticum aestivum* L.), and maize *(Zea mays* L.). Chromosomal localization of *Malus sieversii PYL* gene family members was performed using TBtools^[38]^. The number of amino acids, molecular mass, isoelectric point, instability index, aliphatic, and hydrophilicity index total mean values of *Malus sieversii PYL* gene family genes were studied by Expasy^[39]^ (https://web.expasy.org/compute_pi/). Subcellular localization of *Malus sieversii* PYL proteins was predicted using BUSCA^[40]^ (http://busca.biocomp.unibo.it). Sequence information of the phylogenetic tree of PYL family members of six plants is indicate in Supplementary File S1. Using MEGA 11^[41]^ software was constructed by maximum likelihood (ML) method by setting bootstrap replicates = 1,000. Phylogenetic trees were landscaped at the ChiPlot^[42]^ website (www.chiplot.online/#). Secondary structure prediction was performed using SOPMA^[43]^ (https://npsa-prabi.ibcp.fr/cgi-bin/npsa_automat.pl?page=npsa_sopma.html), and protein 3D structure was performed using RCSB PDB^[44]^ (www.rcsb.org).

### Analysis of the structure and cis-regulatory elements of *PYL* gene in *Malus sieversii*

Conserved structural domains of PYL protein family members were analyzed using the Batch-CD-Search function of NCBI (www.ncbi.nlm.nih.gov/Structure/bwrpsb/bwrpsb.cgi), visualized by the ChiPlot website. The motifs of PYL family proteins were explored using Multiple Em for Motif Elimination (MEME) Version 5.5.7 (https://meme-suite.org/meme/) with the maximum number of motifs identified being 20 and other parameters set to default values. The exon-intron structure of each PYL was identified using GSDS 2.0^[45]^ (http://gsds.gao-lab.org/). The structure of key conserved regions at the functional level of the PYL proteins was identified and the range of conservativeness of each amino acid residue position was calculated using ConSurf Server^[46]^ (http://consurf.tau.ac.il) and classified into three classes: 'variable', 'average', and 'conserved'. The 2,000 bp upstream promoter sequence of the *PYL* gene was obtained in TBtools V2.042 using the GFF3 Sequence Extractor submenu. The obtained file was submitted to PlantCARE^[47]^ (http://bioinformatics.psb.ugent.be/webtools/plantcare/html/) to analyze the *cis*-regulatory elements. The *cis*-elements were also visualized and mapped using R language.

### *PYL* gene collinearity

BLAST alignment and interspecific collinearity analyses of *Malus sieversii* and apple (*Malus domestica*) genome files were performed using Tbtools and MCScanx^[48]^.

### Protein-protein interaction network analysis

To systematically investigate PYL protein interactions, we generated protein-protein interaction (PPI) networks using apple PYL immediate homologous proteins. The interactions of MsPYL proteins were predicted using the STRING database^[49]^ (www.string-db.org; medium confidence level 0.400). PPI networks and node network graphs were constructed and visualized using Cytoscape 3.9.1 software (National Institute of General Medical Sciences (NIGMS))^[50]^.

### Expression analysis of *PYL* gene in *Malus sieversii*

Total RNA was extracted from *Malus sieversii* seeds at five different stratification periods using the TRIzol method, followed by quality assessment. RNA-seq libraries were prepared and subjected to PE150 sequencing on the Illumina^[51]^ platform. Expression profiles of *PYL* gene family members were obtained across different low-temperature sand stratification periods. Publicly available organ-specific expression data were retrieved from the NCBI GEO database (accession: GSE42873)^[52]^, covering the major apple organ (flower, fruit, seed, root, stem, and leaf). Gene expression heatmaps for *PYL* genes across different time points and organs were generated using ChiPlot. Quantitative RT-PCR was as follows: 2 × S6 Universal SYBR qPCR Mix 5 μL, cDNA 1 μL, forward and reverse primers 0.2 μL each, and ddH_2_O to 16.4 μL. Thermocycling conditions were as follows: pre-denaturation at 95 °C for 30 s; three-step amplification at 95 °C/10 s, 60 °C/15 s, and 72 °C/30 s; 40 cycles; lysis 95 °C/10 s; 60 °C/60 s; 95 °C/15 s; cooling 37 °C/30 s. *β-actin* served as the internal reference gene. Relative expression was calculated using the 2^−ΔΔCᴛ^ method^[53]^. Three technical replicates were performed per sample. All primers (Supplementary Table S1) were designed using Primer Premier 5.

### Differentially expressed gene analysis

Gene expression levels were quantified as fragments per kilobase of transcript sequence per million base pairs sequenced (FPKM). Differentially expressed genes (DEGs) were performed using DESeq2, and false discovery rate (FDR) < 0.05 and multiplicity of differences |log2(Fold Change)| ≥ 2^[54]^ were selected as the screening criteria. We conducted functional enrichment analysis of the DEGs using Gene Ontology (GO) (http://geneontology.org), and the Kyoto encyclopedia of genes and genomes (KEGG) databases^[55]^.

### *MsPYL8* cloning and *Arabidopsis* transformation

Designed gene-specific primers based on the coding sequence of *MsPYL8*, amplifying and cloning the *MsPYL8* fragment, ligating the purified PCR product into the pART-CAM-EGFP overexpression vector using *Xho*I and *Eco*RI sites. The overexpression vector was introduced into wild-type (Col-0) *Arabidopsis thaliana* using the *Agrobacterium tumefaciens*-mediated floral dip transformation^[56,57]^. Positive transgenic plants were selected and grown to obtain homozygous T3 generation. Following surface sterilization, both wild-type and the T3-generation transgenic *Arabidopsis* *thaliana* seeds were treated sequentially with: (1) 75% ethanol for 1 min, (2) two rinses with double-distilled water, (3) 8% sodium hypochlorite for 10 min, and (4) five final rinses with double-distilled water. Approximately 50 seeds were sown on either control MS medium without (ABA-free) or MS medium supplemented with 0.3, 0.5, and 0.7 μM ABA, and incubated at 23 °C under a 16 h light/8 h dark photoperiod. For strain phenotypic measurements, 7-day-old seedlings of both Col-0 and *PYL8* transgenic lines (germinated and grown as on MS medium) were transferred to nutrient soil and cultivated for an additional 21 days. Primary root length and total rosette leaf area were quantified using ImageJ^[58]^, with three biological replicates per treatment.

### Yeast two-hybrid assay

A Y2H assay was performed to screen for proteins interacting with the MsPYL8 protein. The coding sequence of the *MsPYL8* was cloned into the pBT3-SUC bait vector using *Xho*I and *Eco*RI, yielding the recombinant plasmid pBT3-SUC-PYL8(PYR1). pBT3-SUC-YF12 + pPR3-N-LOC101266657 served as the positive control, while pBT3-SUC-PYL8(PYR1) + pPR3-N (empty vector) was used as the negative control. For receptor preparation, the pBT3-SUC-PYL8(PYR1) bait plasmid was transformed into yeast strain NMY51. The transformants were plated on SD-TL medium and incubated at 30 °C for 3−5 d to assess potential transcriptional self-activating of pBT3-SUC-PYL8(PYR1). Subsequently, yeast cells harboring pBT3-SUC-PYL8(PYR1) were mated with the pPR3-N membrane cDNA library and plated on three selective media (SD-TL, SD-TLHA + 20 mM 3AT and SD-TLHA + 20 mM 3AT + X-gal plates). All plated were incubated at 30 °C for 3−5 d to select for interacting clones. Putative positive colonies showing interaction with MsPYL8 were verified, and the corresponding prey plasmids were amplified from yeast cells for DNA sequencing. Relevant primer sequences are listed in Supplementary Table S1.

### Point-to-point validation

The yeast plasmids containing candidate interacting proteins were extracted and transformed into *Escherichia coli* DH5α cells for plasmid amplification. The yeast transformation reaction is shown in Supplementary Table S2. Detection of pBT3-SUC-LOC103406509 and pBT3-SUC-LOC103445100 was carried out in the yeast for the presence of transcriptional self-activating activity. Verified single colonies from both the experimental group and the positive control were resuspended in 2 mL of ddH_2_O, and adjusted OD_600_ = 0.002. The concentration was set to three gradients of 10^0^, 10^−1^, and 10^−2^, and sucked up 10 μL for spot plate experiments, and then spotted onto selective gradient media. Three technical replicates were performed per dilution, with plates subsequently incubated at 30 °C for 3−5 d.

### Subcellular localization analysis

Designed cloning primers with *Xho*I and *Eco*RI restriction sites flanking the *MsPYL8* coding sequence. Cloning primers *MsPYL8-pART-CAM-EGFP*-F: 5'-TTGGAGAGGACACGCTCGAGATGGAGA AAGGTGAGGGCTCAATG-3' and *MsPYL8-pART-CAM-EGFP*-R 5'-CCCTTGCTCACCATGAATTCGCATCCACCGTCGCCCG-3'. Amplified *MsPYL8* CDS and performed homologous recombination with linearized pART-CAM-EGFP vector. Transformed the recombinant plasmid into *E. coli* DH5α cells. Verified construct by plasmid extraction and sequencing. Next, the plasmid was transferred to *A. tumefaciens* GV3101. Then, empty pART-CAM-EGFP or *MsPYL8-pART-CAM-EGFP* GV3101 were instantly infiltrated the strain into 5-week-old *Nicotiana benthamiana* leaves. After the infiltrated *N. benthamiana* was cultured in the dark for 12 h and then in low light incubated for 2−3 d, the fluorescence signals were captured with a confocal laser-scanning microscope (Olympus).

### BiFC assay

The full-length coding sequences of *MsPYL8*, *MsLEA* and *MsLTI* were ligated into pCAMBIA1300-nYFP and pCAMBIA1300-cYFP vectors, with the enzyme cleavage site of *Kpn*I, respectively. The recombinant plasmids were transformed into *E. coli* and transferred to *A. tumefaciens* strain GV3101, and the leaves of *N. benthamiana* were then infiltrated with equal amounts of successfully expressed *A. tumefaciens*. The fluorescence signals were detected under a confocal laser scanning microscope after 2−3 d of infiltration.

### Statistical analyses

All experiment data statistics were evaluated using SPSS 25.0. Significant difference test between WT and *MsPYL8* transgenic strains were determined by Student's *t*-test, with at least three biological replicates.

## Results

### Identification and sequence characterization of *PYL* genes in *Malus sieversii*

**Figure 1:**
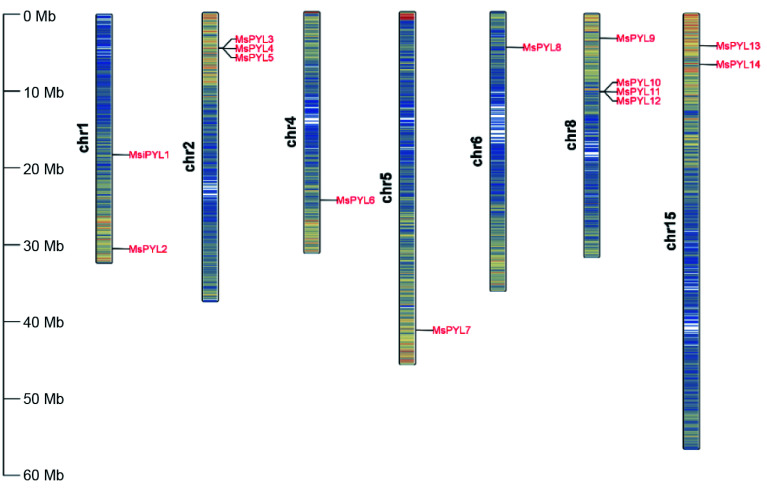
Chromosomal locations of *MsPYLs* in *Malus sieversii*, the scale units on the left are in mega bases.

Genome-wide analysis identified 14 *PYL* genes in *Malus sieversii* based on HMMER's PYL (PF10604). To facilitate the description of these 14 *MsPYL* genes, they were named *MsPYL1*-*MsPYL14* based on their chromosomal locations, and the 14 *MsPYL* genes were unevenly distributed on seven chromosomes, with 10 chromosomes having no members of the family distributed. Among them, they were more distributed on chromosomes 2 and 8, with three and four *MsPYL* genes, respectively. Two were distributed on each of chromosomes 1 and 15, and one *MsPYL* gene was distributed on each of chromosomes 4, 5, and 6 (Fig. 1). Physicochemical analysis of the properties of MsPYL proteins such as amino acid number (aa), molecular mass (MW), isoelectric point (pI), and instability index (Table 1) demonstrated that the number of amino acid residues of the 14 MsPYL proteins ranged from 116 (MsPYL4) to 419 (MsPYL10) and the molecular weight of the protein ranged from 12,843.54 Da (MsPYL4) to 47,701.29 Da (MsPYL10), theoretical isoelectric points ranged from 4.62 (MsPYL12) to 9.79 (MsPYL14), instability coefficients ranged from 24.1 (MsPYL4) to 71.727 (MsPYL14), aliphatic indices ranged from 70.52 (MsPYL4) to 94.39 (MsPYL2), hydrophilicity properties ranged from −0.686 (MsPYL14) to −0.166 (MsPYL11). The hydrophilicity values of all 14 MsPYL proteins were below 0, indicating that all MsPYL proteins are hydrophilic proteins. Subcellular localization predictions demonstrated that 10 MsPYL proteins were localized in the nucleus; MsPYL5 and MsPYL6 were localized in chloroplasts, MsPYL3 and MsPYL4 were localized in the cytoplasm, suggesting that they mainly function in the nucleus.

**Table 1:** Sequence characteristics of 14 MsPYLs in *Malus sieversii*.

Gene name	Genome ID	Protein (aa)	MW (Da)	pI	Instability index	Aliphatic index	GRAVY	Sucellular localization
MsPYL1	Msi_01g007840	184	20,798.62	6.45	51.47	90.98	−0.421	Nucleus
MsPYL2	Msi_01g019270	198	21,804.57	5.97	48.86	94.39	−0.363	Nucleus
MsPYL3	Msi_02g005520	168	18,791.41	4.76	24.23	81.19	−0.245	Cytoplasm
MsPYL4	Msi_02g005570	116	12,843.54	5.15	24.1	70.52	−0.459	Cytoplasm
MsPYL5	Msi_02g005590	160	17,906.48	6.72	38.6	82.81	−0.256	Chloroplast
MsPYL6	Msi_04g014910	250	26,913.81	6.37	47.15	75.2	−0.383	Chloroplast
MsPYL7	Msi_05g028700	185	20,125.74	5.68	49	84.22	−0.223	Nucleus
MsPYL8	Msi_06g003270	206	22,911.59	5.26	42.23	76.55	−0.433	Nucleus
MsPYL9	Msi_08g003790	201	22,120.78	5.02	42.73	80.9	−0.345	Nucleus
MsPYL10	Msi_08g010820	419	47,701.29	6.43	30.5	78.57	−0.342	Nucleus
MsPYL11	Msi_08g010830	194	21,812.06	4.92	30.59	86.86	−0.166	Nucleus
MsPYL12	Msi_08g010850	166	18,336.71	4.62	43.25	74.7	−0.337	Nucleus
MsPYL13	Msi_15g006040	202	22,038.77	5.2	33.84	82.43	−0.309	Nucleus
MsPYL14	Msi_15g009000	294	33,320.02	9.79	71.72	74.69	−0.686	Nucleus

**Figure 2:**
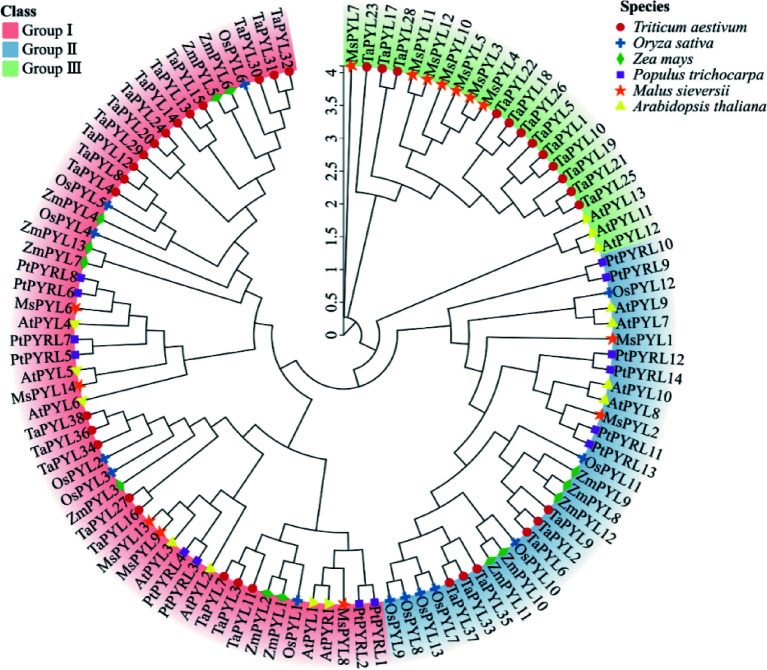
Phylogenetic relationships of PYL proteins *Malus sieversii*, *Triticum aestivum*, *Oryza sativa*, *Zea mays*, *Arabidopsis thaliana,* and *Populus trichocarpa*. Different colours show the three groups (I, II, III) of PYL. Thirty-eight wheat PYLs are represented by red circles, 13 rice PYLs are represented by blue crosses, 13 maize PYLs are represented by green diamonds, 14 *Populus trichocarpa* PYLs are represented by purple rectangles, 14 *Malus sieversii* PYLs are represented by orange pentagrams, and 14 *Arabidopsis thaliana* PYLs are represented by yellow triangles.

Rooted maximum likelihood (ML) phylogenetic trees were constructed using all PYL protein sequences of six plants, including family members from the monocotyledonous plants such as wheat, maize, and rice, and dicotyledonous model plants such as *Arabidopsis*, *P. trichocarpa*, and *M. sieversii*. Sequences were divided into three groups based on the topology of the phylogenetic tree. The first group consisted of 20 dicotyledonous and 34 monocotyledonous plant PYL proteins, including five *M. sieversii* MsPYL6, MsPYL8−9, and MsPYL13−14, seven *Arabidopsis* AtPYR1 and AtPYL1−6, and eight *P. trichocarpa* PtPYRL1−8 proteins. The second group contained 12 dicotyledonous and 18 monocotyledonous plant PYL protein members, including two *M. sieversii* MsPYL1 and MsPYL2, four *Arabidopsis* AtPYL7−10, and six *P. trichocarpa* PtPYRL9−14 proteins. The third group included 10 dicotyledonous and 12 monocotyledonous PYL members, including seven *M. sieversii* MsPYL3−5, MsPYL7, and MsPYL10−12, three *Arabidopsis* AtPYL11−13 proteins, and 12 TaPYL proteins in monocotyledonous plants (Fig. 2). Phylogenetic tree analyses demonstrated that *M. sieversii* was more closely related to *P. trichocarpa* and *Arabidopsis*, and more distantly related to maize and rice, which might be related to the fact that maize and rice are monocotyledons, whereas *M.sieversii* is a dicotyledonous plant with *P.trichocarpa* and *Arabidopsis*. Protein secondary structure demonstrated that the 14 MsPYLs were mainly composed of alpha helix and random coil (Supplementary Table S3). *M. sieversii* MsPYL protein homology modelling selected various templates based on homology scores, including 10 templates 5GWO, 5GTF, 4JDL, 3OQU, 5GTE, 5GTG, 3KDH, 3KDI, 6IES, and 5MOA (Supplementary Fig. S1).

### Analysis of the structure and cis-regulatory elements of *PYL* gene in *Malus sieversii*

All 14 identified MsPYL proteins contained the characteristic PYR_PYL_RCAR-like domain (cd07821), a member of the SRPBCC superfamily (Supplementary Fig. S2b). Twenty conserved motifs among the MsPYL proteins, named Motifs 1−20. Each MsPYL protein contained 5−12 of these motifs (Supplementary Fig. S2c), with motif lengths ranging from 6−41 amino acids (Table 2). The presence of the motifs of MsPYL proteins in the same phylogenetic group was similar, with MsPYL1, 2 shared Motif 1, 2, 3, 4, 5, and 12, MsPYL3, MsPYL4−5 contained Motif 1, 5, and 7, MsPYL6−9 and MsPYL13 shared Motifs 1−5, but MsPYL8−9 and MsPYL13 have Motif 9, MsPYL9 and MsPYL13 have Motif 10, 11, and MsPYL10 uniquely possessed Motif 12. Comparative analysis of exon-intron structures revealed significant diversity among *MsPYL* genes. These structural variations provide important insights into evolutionary relationships within this gene family. Structural analysis of 14 *MsPYL* gene demonstrated that the number of introns in *MsPYL* genes ranged from 0 to 4, and exon numbers ranged from 1 to 5. The majority of *MsPYL* genes exhibited a simple single-exon structure, while specific members showed more complex architectures. *MsPYL6* and *MsPYL11* contained two exons and one intron, *MsPYL1*, *MsPYL2,* and *MsPYL14* contain three exons and two introns, while *MsPYL10* contains five exons and four introns (Supplementary Fig. S2d). Some motifs were identified as evolutionarily conserved, and several variable regions were detected in PYL proteins, with amino acid lengths ranging from 2−16 (Supplementary Fig. S2e). In addition, the discovery of evolutionarily 'conserved' and 'variable' residues in PYL proteins can help to gain insight into the function and evolutionary dynamics of PYL proteins in plants.

**Table 2:** The sequences of *MsPYL* genes identified by the MEME.

Motif	Width	Motif sequences
1	41	PANTSTERLELJDDEKHILSYSIIGGNHRLNNYRSTTTVVP
2	23	VWPLVRDFDNPHKYKPFLKSCHV
3	41	GTVVIESYVVDIPEGNTKEDTCLFVDTVJQLNLKSLAAVAE
4	29	YIDRYHKHEPSPNQCTSLLVQKIEAPVHL
5	15	GGVGSIREVAVVSGL
6	21	ZKQLKWEGKASAELKGTAAEQ
7	11	DPKSDQNQDQE
8	15	DFFGFHKWFPTLAPC
9	19	MDAGHAPPYGLTLAEFSEL
10	11	FVNCDGEEGDN
11	10	HGNTGGGHDQ
12	11	RLAVQDRTEPI
13	6	MEQHWD
14	14	CVIEWFIEVEPVEG
15	13	GVPSQPCVIRYCA
16	6	GCRIEW
17	7	MPLIPVN
18	6	QDQNQK
19	12	PGCIRQCPFFKI
20	6	HPTNQN

**Figure 3:**
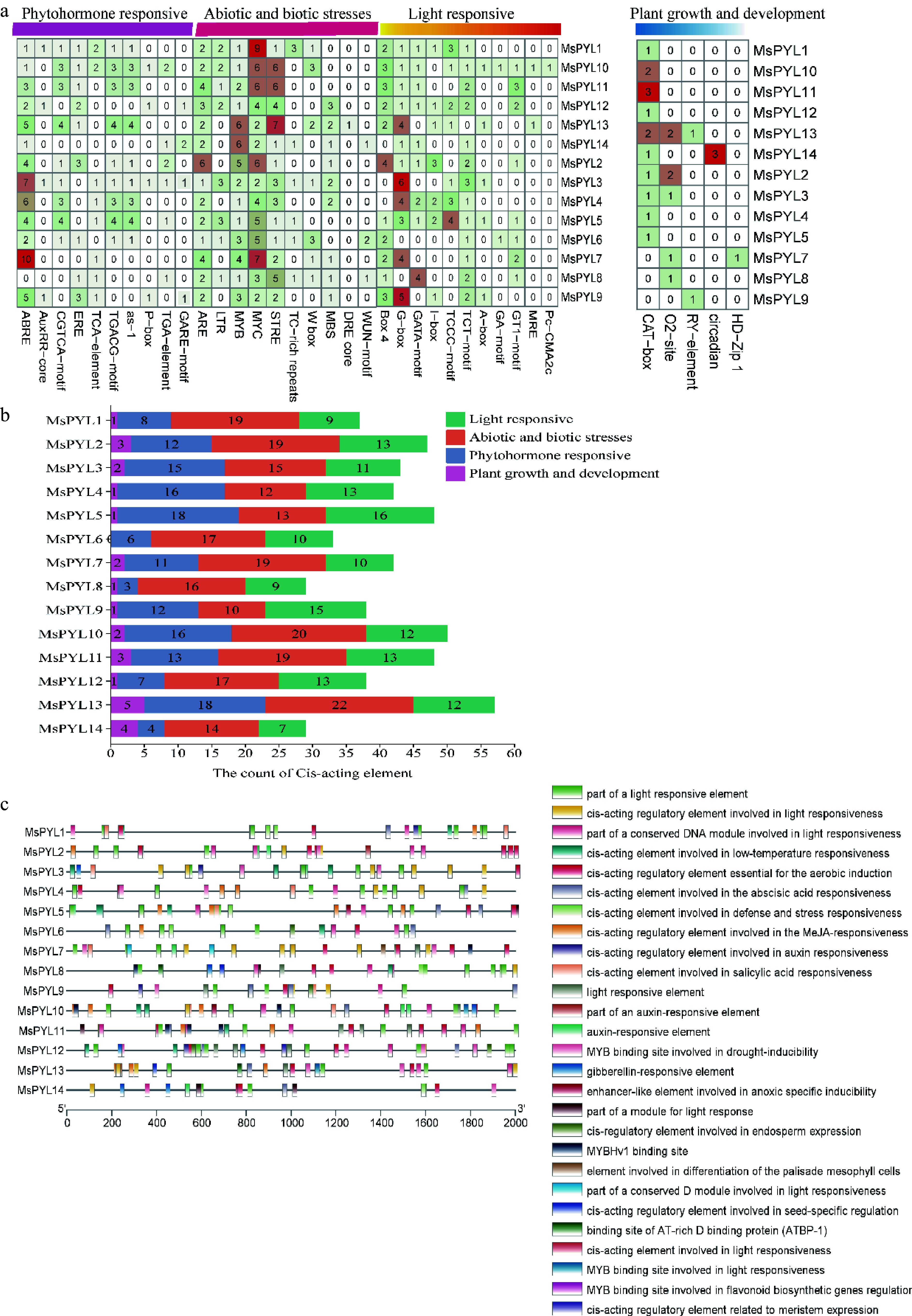
Cis-acting elements on the promoter of the *MsPYL* gene. (a) Four types of *cis*-acting elements. (b) Statistics of the total number of cis-acting elements. (c) Distribution of cis-acting elements: different colour modules on the right represent different cis-acting elements. ABRE, ABA responsive motif; TGACG motif, and CGTCA motif, MeJA responsive motif.

*Cis*-acting element analysis reflects promoter involvement in plant hormone regulation and abiotic stress response. *MsPYL* genes promoters identified 36 cis-acting elements, which were categorized into four types: phytohormone responsive, abiotic and biotic stress response, light response, and plant growth and development (Fig. 3a & c). Eleven phytohormone responsive cis-acting elements were detected, including abscisic acid responsiveness elements (ABRE), growth hormone-responsive elements (AuxRR-core, as-1, TGA-element), ethylene-responsive elements (ERE), salicylic acid-responsive element (TCA-element), methyl jasmonate-responsive (MeJA) element (CGTCA-motif, TGACG-motif), and gibberellin-responsive elements (GARE-motif, P-box). Additionally, 10 abiotic and biotic stress-responsive cis-acting elements were identified, associated with anaerobic (ARE), cold stress (LTR), heat shock (STRE), defense stress (TC-rich repeat), and drought (MYB binding site, MBS), fungus (W-Box), water deficit stress (DRE core) and damage-induced response (WUN-motif). Notably, the promoters of most *MsPYL* genes were found to contain ABRE, for phytohormone response, the ABRE motif related to abscisic acid (ABA) as well as the TGACG-motif and CGTCA-motif related to the methyl jasmonate (MeJA) were mainly enriched. For abiotic and biotic stress response, all *MsPYL* promoters harbored ARE, MYB, and MYC elements and the cis-acting element number statistics found that *MsPYL6* had no plant growth and developmental types, *MsPYL8* and *MsPYL14* had the fewest (28 elements); *MsPYL13* contained the highest number (57 elements) (Fig. 3b). These results concluded that potential role of *PYL* genes in the circadian rhythm, photosynthesis, ABA-mediated stomatal regulation, MeJA-mediated anthocyanin biosynthesis, abiotic and biotic stresses. Distribution of cis-acting elements of *MsPYL* genes (Fig. 3c), these results indicated that the expression of *MsPYL* genes family members was most likely induced by abiotic stresses, such as abscisic acid, drought, and cold temperatures, suggesting that the *PYL* genes family of *M. sieversii* may have an influence hormone response as well as abiotic stresses.

### *PYL* gene collinearity

**Figure 4:**
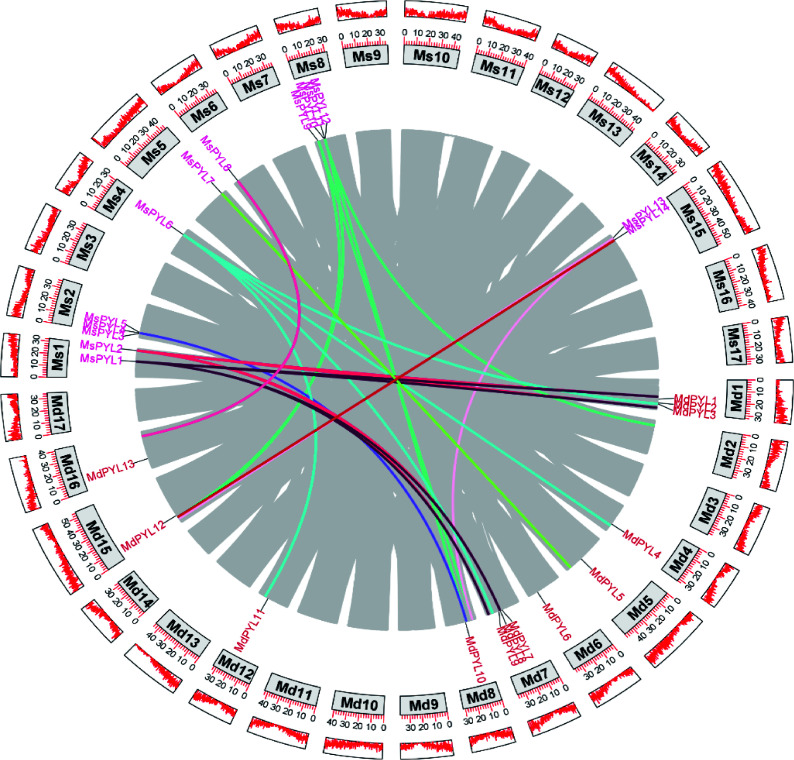
Genome-wide collinearity pairs analysis between the *Malus sieversii* and *Malus domestica PYL* gene families. Different colours represent different *PYL* gene collinearity pairs.

Selection of different species of *Malus domestica* of the same genus for collinearity analysis, genomic collinearity analysis between 14 *MsPYL* genes and 13 *MdPYL* genes was investigated using MCScanx, and five collinearity pairs were identified in the *Malus sieversii* and apple genomes: *MsPYL1*−*MdPYL8*, *MsPYL2*−*MdPYL8*, *MsPYL5*−*MdPYL10*, *MsPYL6*−*MdPYL3*, *MsPYL−MdPYL10* (Fig. 4), indicating that segmental duplication events were pivotal for the amplification of *MsPYL* genes during evolution.

### Protein-protein interaction network analysis

The construction of protein-protein interaction networks helps to find dynamic regulatory networks between biomolecules. The predicted results showed that *Malus sieversii* PYL8 protein interacted with 10 PP2C phosphatases, which subsequently associates with serine/lysine protein kinases (SAPK) and SnRK2, forming a canonical ABA signal transduction cascade. In addition, PYL8 protein interacted with low-temperature-induced 65 kDa proteins-like (LTI-like), and LTI-like interacted with late embryogenesis abundant (LEA, LEA29-like) and cold shock proteins (CS120-like) (Supplementary Fig. S3a & b). Subsequently, GO functional analysis was performed, and among the molecular function aspects, hormone binding (GO:0042562), abscisic acid binding (GO:0010427), protein phosphatase inhibitor activity (GO:0004864), protein phosphatase regulatory activity (GO:0019888), signaling receptor activity (GO:0038023). In the biological process category, signal transduction (GO:0007165), response to hormones (GO:0009725), abscisic acid response (GO:0009737), abscisic acid-activated signaling pathway (GO:0009738), regulation of phosphatase activity (GO:0010921), and phosphoprotein phosphatase activity (GO:0043666), cellular responses to abscisic acid stimulation response (GO:0071215) revealed the characterization of PYL proteins as abscisic acid receptors. Among the cellular component categories, nucleus (GO:0005634), cytoplasm (GO:0005737), organelles (GO:0043226), intracellular anatomical structures (GO:0005622), and obsolete intracellular part (GO:0044424) (Supplementary Fig. S3c). The molecular functions of the PYL proteins are actively involved in abscisic acid binding, and these findings highlight the importance of the role of PYL proteins in abscisic acid signaling as ABA receptors in the regulation of seed dormancy and germination.

### Expression analysis of *PYL* genes in *Malus sieversii*

**Figure 5:**
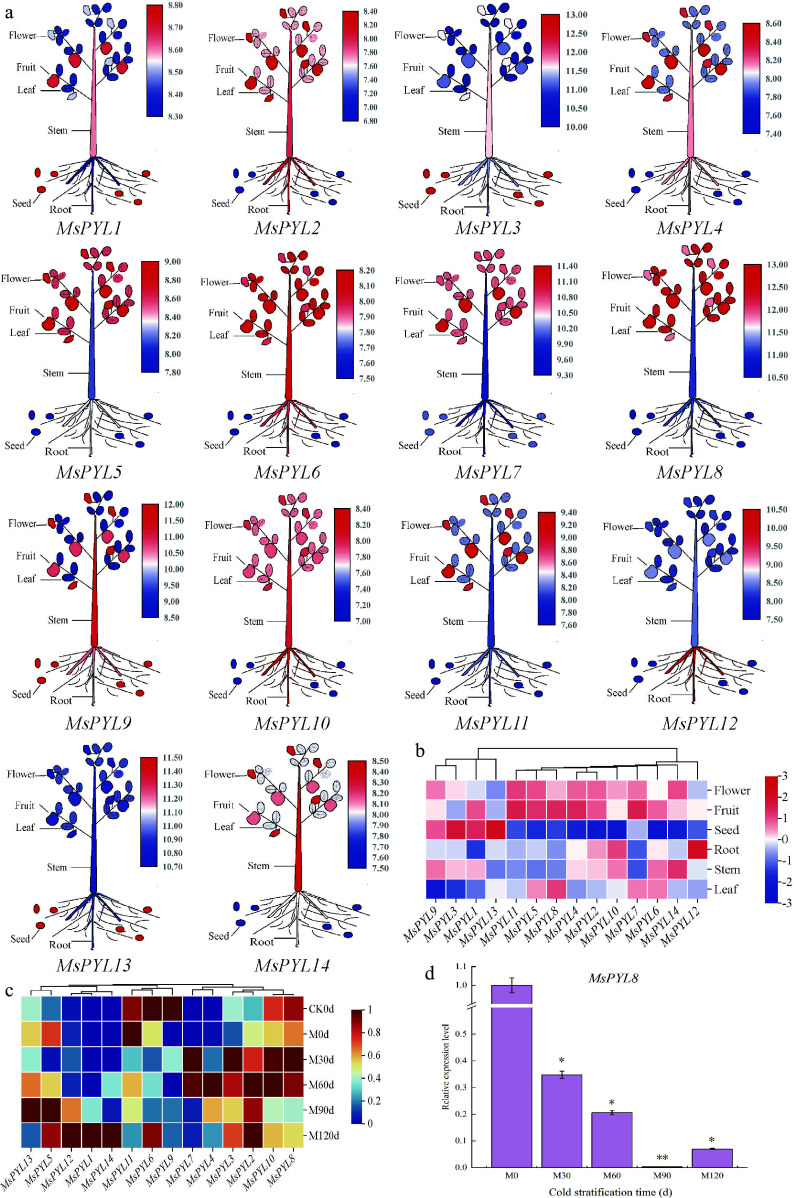
*PYL* gene expression in *Malus sieversii*. (a) The expression pattern of the *Malus sieversii* *PYL* gene in six organs (seeds, roots, stems, leaves, mature fruits, and flowers) is shown in the form of a cartoon diagram of plant simulation. Red colour indicates high expression and blue colour indicates low expression. (b) Heat map showing the expression of *PYL* gene in six organs. (c) Heatmap demonstrating the expression pattern of *PYL* genes in seeds at different periods of low-temperature stratification. (d) *MsPYL8* was analyzed by qRT-PCR. Error lines indicate standard deviation, and the values in the graphs are the mean ± standard deviation (SD) of three independent biological replicates. Student's *t*-test asterisks indicate significant up- or down-regulation of the *MsPYL8* compared to the un-stratified treatment (* *p* < 0.05, ** *p* < 0.01).

The expression characteristics of genes in different organs of plants can well reveal the biological functions they play during growth and development. Transcriptome analysis demonstrated distinct spatiotemporal expression patterns of the 14 *MsPYL* genes across six organ systems (seeds, roots, stems, leaves, flowers, and fruits) (Fig. 5a & b; Supplementary Table S4). *MsPYL2*, *4*, *5*, *6*, *7*, *8*, *10*, *11*, and *14* had high expression levels in flowers and fruits, but relatively low expression levels in seeds. *MsPYL13* exhibited high expression in seeds, with *MsPYL3*, *MsPYL1,* and *MsPYL9*, while *MsPYL12* showed the highest expression in roots, *MsPYL10* and *MsPYL2*. *MsPYL6* had higher expression levels in stems and leaves, and *MsPYL12* had higher expression only in roots and lower expression in other organs. *MsPYL5*, *MsPYL6*, *MsPYL7,* and *MsPYL8* had higher expression levels in leaves than other genes. The above results indicate that *MsPYL* genes play different degrees of roles in participating in the growth and development process.

The transcriptome results of seeds treated with 4 °C low temperature sand stratification showed that *MsPYL2*, *MsPYL3*, *MsPYL4,* and *MsPYL7* were up-regulated, reaching peak expression levels at 30 d or 60 d. *MsPYL1*, *MsPYL5*, *MsPYL12*, *MsPYL13,* and *MsPYL14* showed up-regulated changes, maintaining high expression levels at 90 or 120 d. The expression of *MsPYL8* and *MsPYL10* was high at 30 and 60 d, followed by a decrease in expression at 90 and 120 d (Fig. 5c). These results demonstrate that *MsPYL8* may play a role in seed dormancy lifting under low-temperature stratification treatments.

During the period from M0 to M60 in the low-temperature sand stratification, the ABA content decreased from 80.22 ng·g^−1^ to 54.55 ng·g^−1^, and reached a minimum value of 43.67 ng·g^−1^ at M120; the seeds started to germinate from M60, and the germination rate was 7%, 95%, and 100% at M60, M90, and M120, respectively, which indicated that the change of ABA content affected the seed germination at the periods of M0 and M30. RT-PCR analysis revealed that *MsPYL8* expression remained relatively low during seed germination under low-temperature sand stratification (Fig. 5d), mirroring the observed decrease in ABA content in *Malus sieversii* seeds, and it is hypothesized that this gene plays a positive regulatory role in response to ABA induction.

### Determination of DEG for low-temperature sand storage stratification processes

Comparative transcriptome analysis identified significant DEG patterns during low-temperature sand stratification of *Malus sieversii* seeds. In M0 d vs CK0 d, we detected 4,547 DEGs (2,559 up-regulated and 1,988 down-regulated) (Supplementary Fig. S4a), with GO enrichment analysis showing predominant involvement in intracellular components (1,500 genes), binding activities, and DNA-binding transcription factor functions (Supplementary Fig. S4b). KEGG pathway analysis further revealed significant enrichment in protein processing within the endoplasmic reticulum (62 genes), spliceosome activity (61 genes), and plant hormone signal transduction (59 genes) (Supplementary Fig. S4c). As stratification progressed, the M30 vs CK30 comparison showed 12,066 DEGs (7,381 up-regulated and 4,685 down-regulated) (Supplementary Fig. S5a). While M60 d vs CK60 d included 12,122 DEGs (7,358 were up-regulated and 4,764 were down-regulated) (Supplementary Fig. S6a). By 90 d (M90 d vs CK90 d), DEG numbers decreased to 9,311 DEGs (5,792 were up-regulated and 3,519 were down-regulated) (Supplementary Fig. S7a). Notably, the M120 d vs CK120 d comparison demonstrated a three-fold increase in DEG quantity relative to M0 vs CK0 (Supplementary Fig. S8a).

Gene ontology enrichment analysis of DEGs was performed, and the biological processes were mainly involved in the response to osmotic stress and water deprivation, abscisic acid response, response to salt stress, transcriptional regulation, regulation of seed germination, and gibberellin response (Supplementary Fig. S6b). Of the cellular components, DEGs were mainly chloroplast stroma, chloroplast envelope, and thylakoid. At the molecular functions, DEGs were mainly related to DNA-binding transcription factor activity, phosphatase activity and protein homodimer activation. Multiple plant hormone-related biological processes remained active and underwent changes during low-temperature sand storage stratification of *Malus sieversii* seeds, suggesting their involvement in seed dormancy release activities. KEGG pathway enrichment analysis showed that the key pathways enriched in seeds during low-temperature sand storage stratification across different periods included plant hormone signaling, the plant MAPK signaling pathway, protein processing in the endoplasmic reticulum, starch, and sucrose metabolism, and glycolysis/glycogenesis (Supplementary Figs S5b, S5c, S6c, S7b, S8c). These findings demonstrated that coordinated activity across these pathways is essential for mediating seed germination under low-temperature sand stratification.

### *PYL8* mediates negative regulation of seed germination by ABA signaling

**Figure 6:**
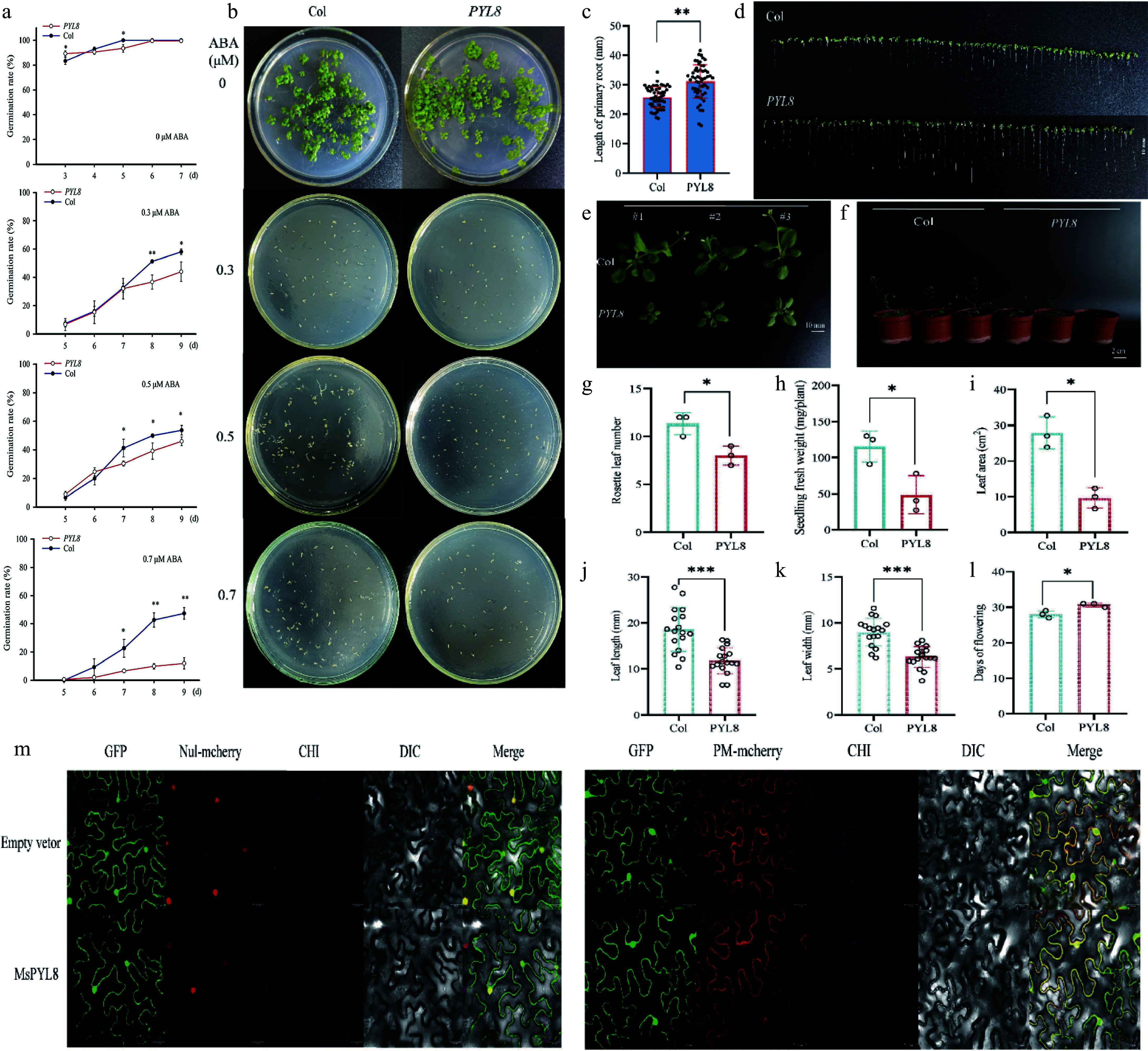
*PYL8* functional validation and PYL8 subcellular localization. (a) Germination rate measurement. Seeds of Col and *PYL8* strains were seeded on Murashige and Skoog (MS) medium containing different concentrations of ABA. Germination was defined as the emergence of embryo growth, with germination rates recorded daily until day 7 (for controls without ABA), or day 9 (for treatments with 0.3, 0.5, and 0.7 μM ABA). Data are means ± SD (n = 3), Student's t-test (* *p* < 0.05) (** *p* < 0.01). (b) Seed germination of *PYL8* strain was inhibited by different concentrations of ABA. Col seeds were cultured on MS medium without ABA (control) for 7 d, and *PYL8* seeds were cultured on MS medium containing different concentrations of ABA (0.3, 0.5, and 0.7 μM ABA) for 12 d. (c) Seven-day root length statistics of Col and *PYL8* strains. Values are means ± SD (n = 56), Student's *t*-test (** *p* < 0.01). (d) Seven-day root length of Col and *PYL8* strains. Scale bar represents 10 mm. (e) Phenotypic analysis of 28-d Col, *PYL8* strains. Col, PYL8 transgenic plants that sprouted and grew on MS medium at 7 d of age were transferred to nutrient soil and continued to grow for 21 d. Scale bar = 10 mm. (f) Flowering phenotypes of 35-d Col, *PYL8* strains. (g) Statistical analysis of *Arabidopsis* rosette leaves. Data are means ± SD (n = 3), Student's *t*-test (* *p* < 0.05). (h) Statistical analysis of fresh weight of plant above-ground parts. Data are means ± SD (n = 3), Student's *t*-test (* *p* < 0.05). (i) Statistical analysis of total plant leaf area. Data are presented as mean ± SD (n = 3), Student's *t*-test (* *p* < 0.05). (j) Statistical analysis of plant leaf length. Data are presented as mean ± SD (n = 17), Student's *t*-test (*** *p* < 0.001). (k) Statistical analysis of plant leaf width. Data are presented as mean ± SD (n = 17), Student's *t*-test (*** *p* < 0.001). (l) Statistical analysis of plant flowering time. Data are presented as mean ± SD (n = 3), Student's *t*-test (* *p* < 0.05). (m) Subcellular localization of MsPYL8 protein in *N. benthamiana* leaves. Scale bars = 25 μm.

The seed germination rates revealed significant differences between *PYL8* and Col at 0 μM ABA concentration on days 3 and 5 (*p* < 0.05), and all the seeds had germinated on day 6. At 0.3 μM ABA concentration, the seed germination rate of Col and *PYL8* was significantly different on day 8 (*p* < 0.01), and the seed germination rate of *PYL8* were about 15% lower than those of Col. At 0.5 μM ABA concentration, the seed germination rates of Col and *PYL8* was different from day 7 onwards (*p* < 0.05), and the seed germination rate of *PYL8* was about 8% lower than that of Col on day 9. At 0.7 μM ABA concentration, the seed germination rates of Col and *PYL8* were different on day 7 (*p* < 0.05), and significantly different on days 8 and 9 (*p* < 0.01), and approximately 35% decrease for *PYL8* than for Col on day 9 (Fig. 6a & b). We measured the primary root lengths of seedlings of the Col and *PYL8* strains (Fig. 6c). Seven-day-old seedlings were grown on MS medium without ABA, and the primary root lengths of *PYL8* strain seedlings were greater than those of Col seedlings, with an increase of the mean length of the *PYL8* primary roots by about 5.4 mm, which was significantly different (*p* < 0.01) (Fig. 6d). Thus, the *PYL8* strain inhibited ABA-mediated seed germination more than the wild type, and overexpression of *PYL8* enhanced ABA signaling and inhibited seed germination. *PYL8* may negatively regulate seed germination through ABA signaling, and *PYL8* transgenic plants increased the length of the primary root in *Arabidopsis*, suggesting that overexpression of *PYL8* reduced the sensitivity of *Arabidopsis* seedlings to ABA.

The 28-d *PYL8* transgenic strain exhibited delayed growth and reduced biomass of aboveground parts compared to Col (Fig. 6e & f). The number of rosette leaves was lower in *PYL8* than in Col (*p* < 0.05) (Fig. 6g), and aboveground fresh weight was 58% lower in *PYL8* (*p* < 0.05) (Fig. 6h). The rosette leaf area of *PYL8* lines was smaller than that of Col (*p* < 0.05), and the rosette leaf area of *PYL8* was reduced by approximately 65% compared with that of Col (Fig. 6i), suggesting that overexpression of *PYL8* had a significant inhibitory effect on *Arabidopsis* growth. The rosette leaf length and width of *PYL8* were reduced by 37% and 30%, respectively, compared with that of Col (*p* < 0.001) (Fig. 6j & k), and flowering was delayed (Fig. 6l). These results indicate that *PYL8* overexpression significantly inhibits *Arabidopsis* growth, leading to a longer growth cycle and markedly reduced aboveground biomass compared to Col.

Additionally, fusion fluorescence signals were observed in both the plasma membrane and the nucleus. This phenomenon effectively demonstrated the expression and functional operation of the MsPYL8 protein in the plasma membrane and nucleus (Fig. 6m).

### Yeast two-hybrid screening for MsPYL8-interacting proteins

The pBT3-SUC-PYL8(PYR1) + pPR3-N transformants failed to grow on plates containing 20 mM 3AT, confirming that full-length MsPYL8 protein lacks self-activating activity (Supplementary Fig. S9a). This result validates its suitability as bait for interaction protein screening. From 1,591 sequences clones, we identified 30 non-repetitive candidate interaction proteins of MsPYL8 (Table 3), including two late embryogenesis abundant proteins, a low-temperature-induced 65 kDa protein-like, 10 ribosomal proteins, and senescence-related proteins. Based on their roles in seed germination and reproductive development, late embryogenesis abundant proteins (LOC103445100) and low-temperature-induced (LOC103406509) proteins were screened as MsPYL8 interacting proteins for further validation. Point-to-point through Y2H assay confirmed the interaction between PYL8 and these proteins. Positive and negative control plasmid-transformed yeast cells were cultured on SD-TLHA + 20 mM 3AT, and SD-TLHA + 20 mM 3AT + X-gal medium, respectively (Supplementary Fig. S9d). The results were consistent with the initial screening, demonstrating that MsPYL8 interacts with MsLEA and MsLTI, respectively. The yeast two-hybrid point-to-point assay was used to further validate the interaction between MsPYL8 and MsLTI in plants. Additionally, we verified the absence of self-activating activity for MsLTI and MsLEA (Supplementary Fig. S9b, c), further supporting the reliability of the Y2H interactions.

**Table 3:** The annotation of the proteins interacting with MsPYL8 by Y2H-seq assay.

No.	Gene ID	Annotation
1	LOC103438579	Cysteine proteinase inhibitor 6-like isoform X2
3	LOC103430899	60S ribosomal protein L21-1
4	LOC103449145	Stress-related protein-like
8	LOC103453156	40S ribosomal protein s3a-2-like
14	LOC103427451	60S ribosomal protein L22-2-like
17	LOC103409643	60S ribosomal protein l7a-1-like
23	LOC114819509	Outer envelope pore protein 16, chloroplastic-like isoform X1
24	LOC103435311	Protein SUPPRESSOR OF FRI 4-like
25	LOC103445100	Late embryogenesis abundant protein
40	LOC126599572	60S acidic ribosomal protein P2-1-like
41	LOC103419916	40S ribosomal protein S4-3
42	LOC103443916	Probable F-actin-capping protein subunit beta
44	LOC114822900	Phytochrome-interacting ankyrin-repeat protein 2-like isoform X1
45	LOC103448729	Transcription elongation factor 1 homolog
48	LOC103450718	60S ribosomal protein L36-3-like
49	LOC103418974	40S ribosomal protein S8
51	LOC103438259	Uncharacterized protein LOC103438259
55	LOC103446105	Obg-like atpase 1
56	LOC103444213	Embryonic protein DC-8-like isoform X1
57	LOC103433780	Uncharacterized protein LOC103433780
69	LOC103432335	60S ribosomal protein L14-1-like
74	LOC103452893	Late embryogenesis abundant protein 41
75	LOC103406509	Low-temperature-induced 65 kDa protein-like
77	LOC103450479	7-methyl-GTP pyrophosphatase
80	LOC126608056	T-complex protein 1 subunit epsilon-like
85	LOC103423984	Translationally-controlled tumor protein homolog
88	LOC126591089	Protein SENESCENCE-ASSOCIATED GENE 21, mitochondrial-like
89	LOC103445970	40S ribosomal protein S4
91	LOC103453729	Uncharacterized LOC103453729
92	LOC103433881	Probable protein disulfide-isomerase A6

**Figure 7:**
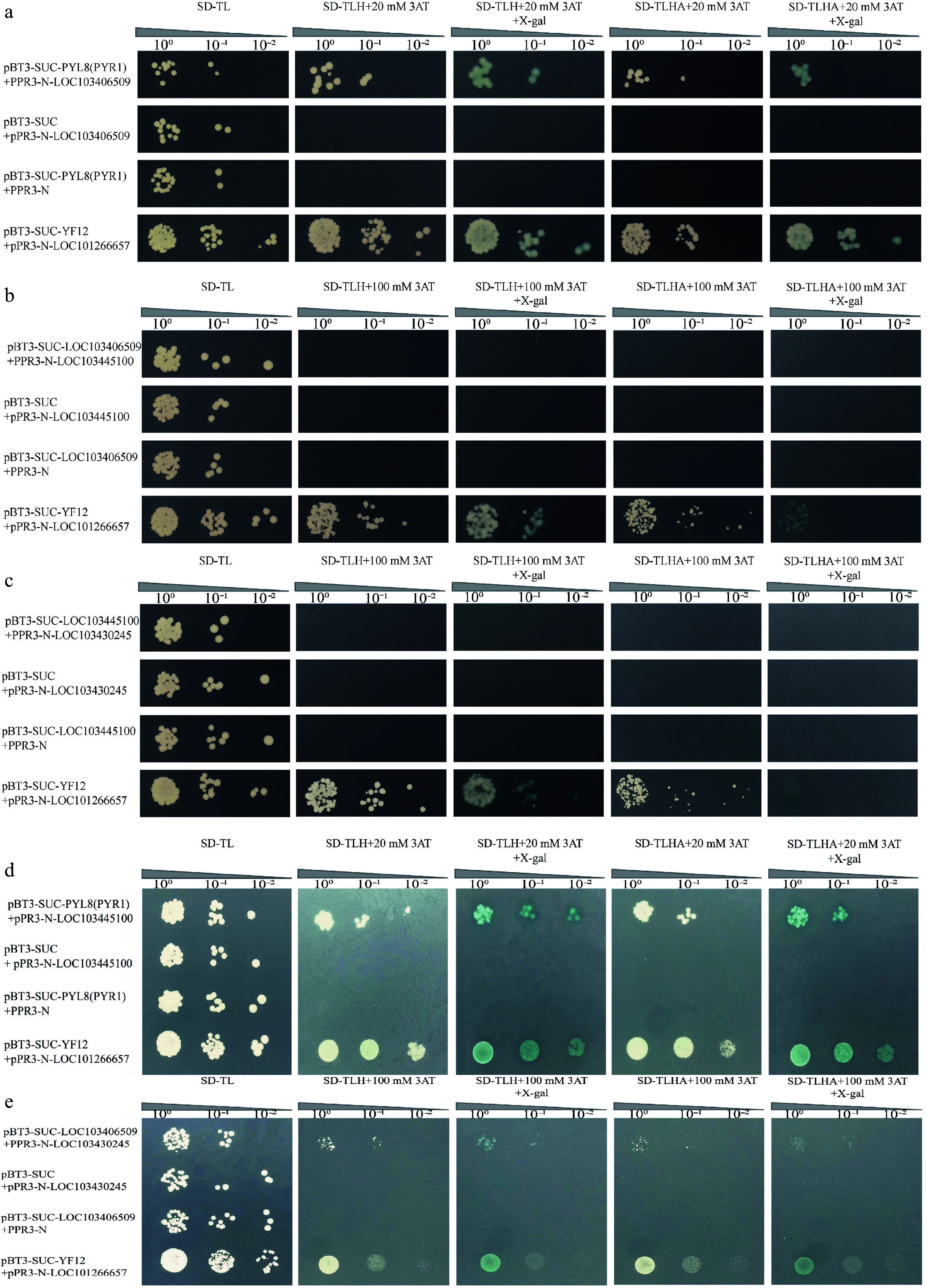
Point-to-point through Y2H assay of MsPYL8 interactions proteins. (a) Positive plasmids were tested with the Y2H assay for point-to-point validation of MsPYL8-MsLTI. pBT3-SUC-PYL8(PYR1) + pPR3-N and pBT3-SUC-LOC103406509 + pPR3-N were the negative controls. (b) Point-to-point validation of the positive plasmid with MsLTI-MsLEA. pBT3-SUC-LOC103406509 + pPR3-N and pBT3-SUC-LOC103445100 + pPR3-N were as negative controls. (c) Point-to-point validation of positive plasmids with MsLEA-MsABI5. (d) Positive plasmids were tested with the Y2H assay for point-to-point validation of MsPYL8-MsLEA. pBT3-SUC-PYL8(PYR1) + pPR3-N and pBT3-SUC-LOC103445100 + pPR3-N were the negative controls. (e) Point-to-point validation of positive plasmids with MsLTI-MsABI5. pBT3-SUC + pPR3-N-LOC103406509, pBT3-SUC + pPR3-N-LOC103430245 were used as negative controls. Spotting dilution gradient was 10^0^, 10^−1^, 10^−2^ from left to right. SD-TL is a SD/-Trp-Leu-deficient plate, SD-TLHA is a SD/-Trp-Leu-His-Ade-deficient plate, where X-gal was added to produce a blue colour.

**Figure 8:**
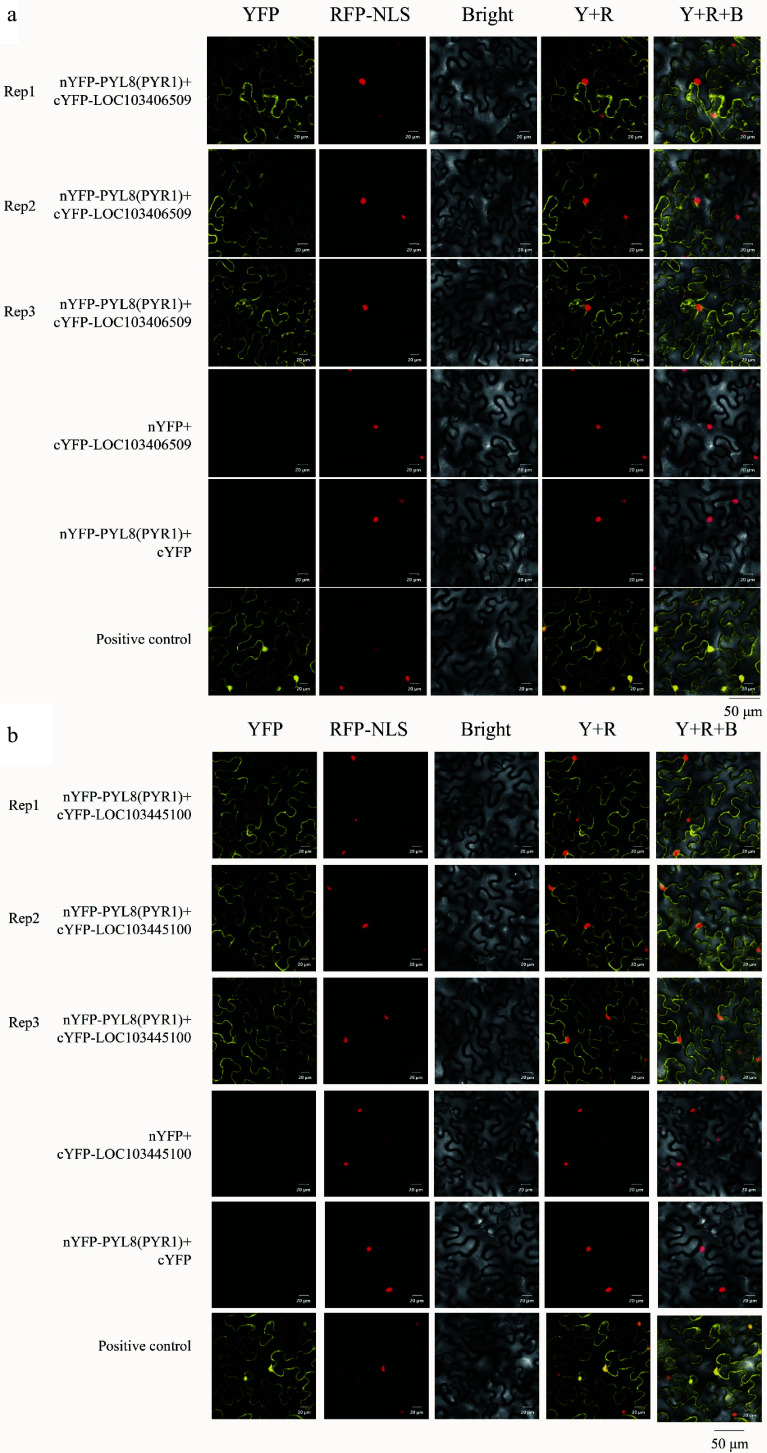
BiFC assay of MsPYL8 interactions proteins. (a) BiFC assay demonstrating the interactions of MsPYL8 with MsLTI at the nucleus and in the cytoplasm in tobacco leaves. (b) BiFC assay demonstrating the interactions of MsPYL8 with MsLEA at the cytoplasm in tobacco leaves. Scale bar = 20 μm.

The experimental group pBT3-SUC-PYL8(PYR1) + pPR3-N-LOC103406509 exhibited normal growth and blue colour on both SD-TLH + 20 mM 3AT + X-gal, SD-TLHA + 20 mM 3AT + X-gal plates. The experimental groups grew normally on SD-TL-deficient plates, and showed blue colour on SD-TLH + 100 mM 3AT, SD-TLH + 100 mM 3AT + X-gal, SD-TLHA + 100 mM 3AT, and SD-TLHA + 100 mM 3AT + X-gal plates did not grow (Fig. 7a−c). This indicates that MsPYL8 interacts with MsLTI and MsLEA in both plants and yeast, MsLTI interacts with MsABI5 (Fig. 7d, e), MsLTI does not interact with MsLEA, and MsLEA does not interact with MsABI5. However, BiFC assays revealed that the MsPYL8 and MsLTI have interactions and may occur at the cytoplasm and in the nucleus (Fig. 8a), and that MsPYL8 and MsLEA have interactions and may occur at the cytoplasm (Fig. 8b).

## Discussion

Abscisic acid plays crucial roles in plant physiology, including seed maturation, stomatal movement, and stress tolerance. The *PYL* gene family has been identified and functionally across diverse plant species, including pattern, economic crops, and medicinal plants. For example, *Arabidopsis thaliana* (14)^[59]^; a total of 21, 20, 40, and 39 *PYL* genes were identified in the diploid progenitor *Gossypium arboretum*, *G. raimondii*, and the tetraploid *G. hirsutum* and *G. barbadense*, respectively^[60]^. Rice (*Oryza sativa*) (13)^[61]^, soybean (*Glycine max*) (24)^[62]^, *Nicotiana benthamiana* (23)^[63]^, white pear (*Pyrus bretschneideri*) (11)^[64]^, 13, 14, and 14 *PYL* genes were identified in *Ipomoea batatas*, *Ipomoea trifida,* and *Ipomoea triloba* of the sweetpotato^[65]^, respectively. Tea tree (*Camellia sinensis*) (20)^[66]^ and Goji (*Lycium barbarum*) (12)^[67]^. Phylogenetic analyses showed that *Malus sieversii* was more closely related to *P. trichocarpa* and *Arabidopsis*, and more distantly related to maize and rice. This phylogenetic clustering correlates with their taxonomic classification, whereas *Malus sieversii* is a dicotyledonous plant together with *P. trichocarpa* and *Arabidopsis*, while maize and rice are monocotyledonous plants. Phylogenetic analyses of the PYL protein sequences revealed sequence differences between monocotyledons and true dicotyledons, which may result in the number of PYL proteins within each plant subfamily and PYL proteins number variation within species. The PYL proteins of *Malus sieversii* may have a similar evolutionary history with the homologous *Arabidopsis* and *P. trichocarpa*.

Conserved motif analyses were conducted to explore the diversity of the *PYL* gene family at the protein level, with each *MsPYL* genes family member varying in the number and composition of motifs, all family members except *MsPYL14* having Motif 1, while all *PYL* gene members except *MsPYL4* having Motif 2. MEME analyzes showed that Motifs 1 and 2 belong to the polyketide cyclase/dehydratase and lipid transport superfamily proteins. Eight *PYL* genes had no introns and only six *PYL* genes had intronic structures. Gilbert^[68]^ illustrated that there is a correlation between the evolution of ancient genes and introns in gene structures. The hypothesis of the origin of introns in gene structure is divided into two types, one is the existence of introns within the gene itself, and the other is the creation of introns during gene replication by transposition. In this study, one intron was present in *MsPYL6* and *MsPYL11*, which were the first genes to appear in evolution, two introns were present in *MsPYL1*, *MsPYL2,* and *MsPYL14*, four introns were present in *MsPYL10*, and the five genes mentioned above appeared subsequently in the process of gene evolution. Intron-derived motifs enhance mRNA accumulation and post-transcriptional regulation of gene expression in both splicing-dependent and splicing-independent ways^[69]^. Six *MsPYL* gene members evolved introns to regulate genes. Analysis of collinearity results comparing genomes on a genome-wide scale revealed that gene duplication events, including polyploidization or result in fragmentation and tandem duplication, serve as the main mechanisms for the generation of new genes and expansion of gene families in plants. These duplicated genes subsequently accumulate mutations, potentially acquiring novel biological functions^[70]^. Functional diversity caused by replication events may lead to changes in gene expression levels and protein properties^[71]^. We also found cis-acting elements involved in abscisic acid, drought, and low temperature in the promoter of the *MsPYL* genes, which demonstrates that the *MsPYL* genes may play a role in the response to plant hormones as well as abiotic stress adaptation.

Most *MsPYL* genes exhibited organ-specific expression patterns, with *MsPYL2*, *4*, *5*, *6*, *7*, *8*, *10*, *11*, *14* showing high expression in both flowers and fruits, but relatively low expression in seeds. *MsPYL5*, *6*, *7,* and *MsPYL8* had higher expression levels in leaves than other genes. Cucumber (*Cucumis sativus* L.) *CsPYL* genes were found to have the same organs-specific expression pattern^[72]^. Rubber (*Hevea brasiliensis*) 14 *HbPYL* genes expression analysis demonstrated that *HbPYL6−14* genes showed higher transcript expression levels in latex compared to other organs (roots, bark, leaves, flowers)^[73]^. *PYL* genes have different functions in plant species, highly emphasizing their key role in plant development. In *Arabidopsis*, PYR/PYL receptors regulate multiple developmental processes including seed germination and establishment, nutritional and reproductive growth, and are involved in ABA-mediated transcriptional responses to seed-based ABA signaling, stomatal aperture and regulatory hormones^[74,75]^. Furthermore, *AtPYL8* and *AtPYL9* interact directly with the auxin-responsive transcription factor *MYB77* to promote lateral root growth by alleviating ABA-mediated inhibition^[18,76]^. CRISPR/Cas9-mediated editing of group I *PYL* genes (*PYL1*-*PYL6* and *PYL12*) in rice was shown to affect seed dormancy and growth regulation, among other aspects^[77]^, highlighting the crucial and multifaceted roles of *PYL* family members in plant development and stress adaptation.

**Figure 9:**
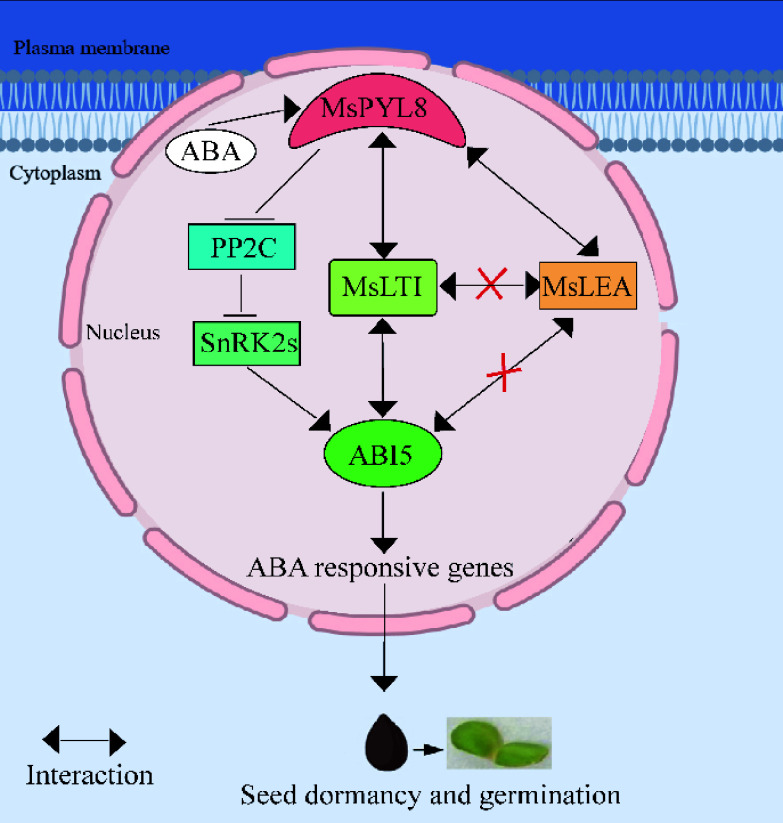
The working model describes the ABA receptor MsPYL8 interaction with MsLTI and MsLEA, MsLTI interacts with MsABI5, and the inhibition of MsPYL8 expression under low-temperature sand stratification to coordinate seed dormancy and germination. MsPYL8 binds to ABA and inhibits the dephosphorylation activity of PP2C, which activates SnRK2s to transmit signals to ABI5, thereby activating the expression of ABA-responsive genes to regulate ABA signal transduction. Arrows, positive regulation; bars, negative regulation.

To further explore the role of *MsPYL8* in ABA-mediated seed germination, transgenic plant lines overexpressing *MsPYL8* were generated through genetic transformation methods. Under ABA treatment, these transgenic plants exhibited a significantly reduced germination rate but enhanced primary root growth compared to the wild type (Fig. 6c & d), indicating that *MsPYL8* functions as a negative regulator of seed germination. The transgenic lines also exhibited delayed growth phenotypes, prolonged flowering time, and reduced aboveground biomass. The processes of protein phosphorylation and dephosphorylation play a crucial role in maintaining the ABA-mediated growth regulatory balance, where PP2Cs serve as core negative regulators by inhibiting SnRK kinases (SnRK1s, SnRK2s, and SnRK3s) in both ABA signaling and abiotic stress responses^[12,78−80]^. ABI5 regulating *PYL11* and *PYL12* expression to modulate ABA-mediated seed germination^[18]^. The dynamic regulation of ABA during seed germination inhibits seed germination and post-germination growth through the ABA receptors PYR/PYL/RCAR and PP2C co-receptors^[23]^. These reports suggest that the interaction between PYL and PP2C is an important pathway for ABA-mediated seed germination. Our study further revealed that MsPYL8 interacts with MsLEA and MsLTI, respectively. LEA is a protein whose expression in embryos increases substantially during late embryogenesis and disappears at the late developmental stage of the seed germination stage^[81]^. Research has indicated that rice OsLEA5 regulates ABA-inhibited germination via reactive oxygen species (ROS) through its interaction with ZFP36 and modulation of ascorbate peroxidase (APX) activity^[82]^. *OsLEA33* affects grain size through reduced brassinosteroid (BR) accumulation and enhanced GA biosynthesis, thereby promoting seed germination^[83]^. Wheat *TaLEA-1A* and its functionally related proteins (ATEM6 and OsEm1) play crucial roles in regulating seed dormancy and germination by modulating ABA and GA pathways^[81]^. Additionally, a low-temperature-induced 65 kDa protein-like, together with four peroxidases (PRX) and other components, mediates responses to both water deficit and hypertonic salinity during seed germination and early seedling growth in *Tamarix hispida*^[84]^. The bZIP transcription factor ABI5 is a positive regulator in ABA-mediated seed germination signaling. Under exogenous ABA treatment, ABA receptor (PYLs) signals to ABI5 during germination via SnRK2s activation. The stability of ABI5 is regulated through multiple post-translational modifications including ubiquitination, SUMOylation, and S-nitrosylation. This regulatory mechanism subsequently activates ABA-responsive gene expression, maintains *PYL* gene expression, and establishes feedback regulation of ABA signal transduction to precisely control seed germination^[18]^. The ABA receptor MsPYL8-mediated regulatory pathway of seed germination is presented in Fig. 9. Based on integrated analysis of our results and existing literature, it is hypothesized that MsPYL8 regulates the seed dormancy lifting pathway of *M. sieversii* as follows: the first one is the canonical ABA-MsPYL8-PP2C-SnRK2s-ABI5 pathway, the second pathway involves low temperature and ABA-induced expression of LTI (a low-temperature-induced protein), which interacts with MsPYL8 following low-temperature stratification treatment. MsLTI also interacts with ABI5, forming an ABA-MsPYL8-MsLTI-ABI5 regulatory axis whereas MsPYL8 serves as the binding hub of this synergistic canonical pathway to control seed dormancy release, the third pathway involves low temperature and ABA-induced expression of late embryogenesis abundant (LEA) protein, which interacts with MsPYL8 but does not interact with ABI5. The next step will focus on functionally validating the interactions of *MsLTI* and *MsLEA* genes, identifying the downstream target genes of *MsLTI* and *MsLEA*, and further elucidating the role of ABA signal transduction pathway in regulating seed dormancy and germination processes.

## Conclusions

Fourteen *PYL* genes were identified from *Malus sieversii* genome, which were classified into three subfamilies, and MsPYL proteins exist PYR_PYL_RCAR_like conserved structural domains. Expression analysis revealed that most of the *MsPYL* genes showed organ-specific patterns, with RNA-seq analysis demonstrating that the 14 *PYL* genes showed differential expression patterns in plant tissue organs. Functional characterization of *MsPYL8* through transgenic *Arabidopsis* overexpression lines showed significant inhibition of seed germination compared to wild-type controls, under increasing ABA concentrations. These transgenic plants exhibited prolonged growth cycles and reduced above-ground biomass. Our findings provide valuable insights into the *PYL* gene family in *Malus sieversii* and lay a foundation for further understanding their hormone signaling processes and biological functions.

## SUPPLEMENTARY DATA

Supplementary data to this article can be found online.

## Data Availability

All data generated or analyzed during this study are included in this published article and its supplementary information files. The tissue-specific expression data that support the findings in the National Center for Biotechnology Information GEO database under the accession number GSE42873.
