# Supplementary material for: Identification of Malus sieversii ABA receptor PYL8 interacting proteome using Y2H-seq
Source: For Res (Fayettev). 2025 Jun 30;5:e012. doi: 10.48130/forres-0025-0012 (PMC12441796; doi:10.48130/forres-0025-0012)
Supplement: Supplementary file 1 — Supplementary data to this article can be found online. [file FR-2025-5-0012-Supplementary.zip › 10.48130_forres-0025-0012-Suppl-FigureS2.pdf]

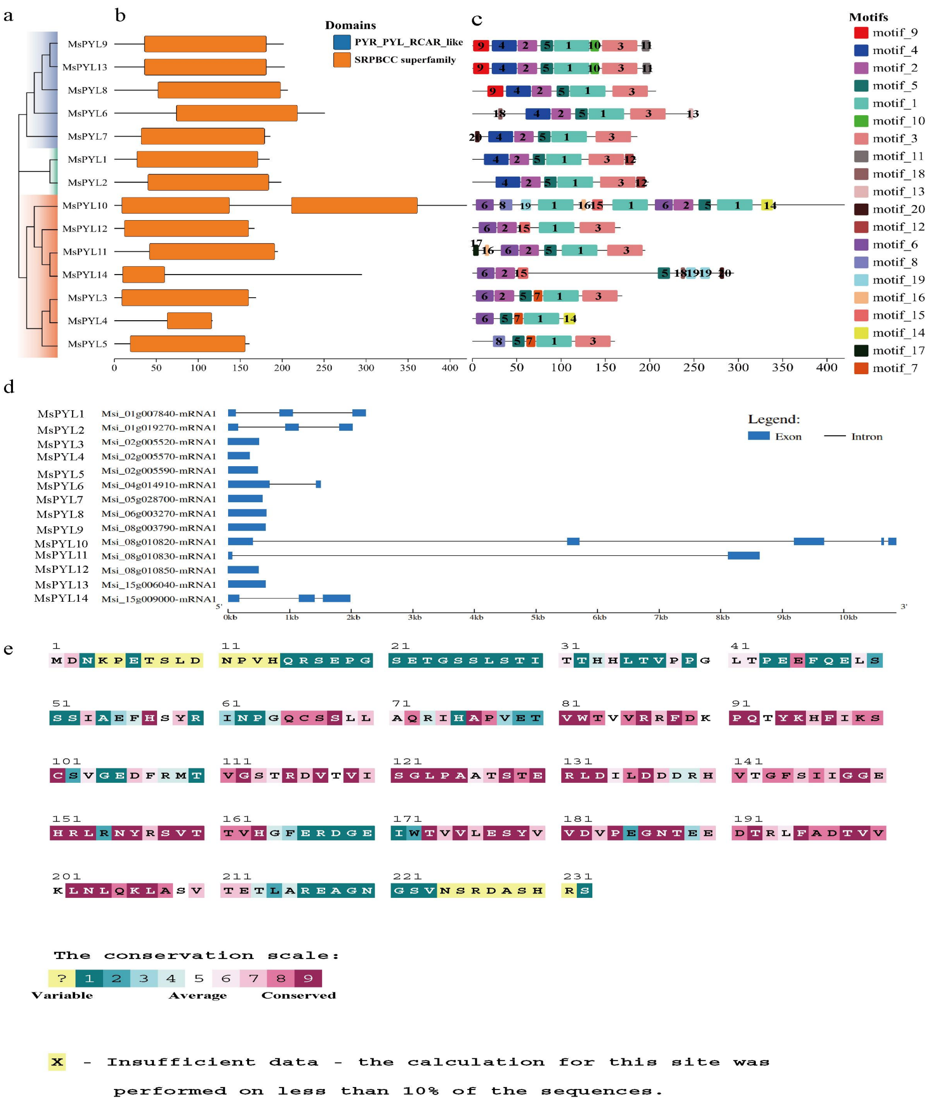

Supplemental Fig.S2 Conserved motifs, gene structure and amino acid sequence conservation of PYL in *Malus sieversii*. (a) Phylogenetic tree. (b) Distribution of conserved domains. (c) Conserved motif distribution. (d) Gene structure: exon-intron distribution map. Blue boxes indicate exons, black lines indicate introns. (e) Identification of amino acid conservation of homologous PYL proteins.
