# Supplementary material for: Identification of Malus sieversii ABA receptor PYL8 interacting proteome using Y2H-seq
Source: For Res (Fayettev). 2025 Jun 30;5:e012. doi: 10.48130/forres-0025-0012 (PMC12441796; doi:10.48130/forres-0025-0012)
Supplement: Supplementary file 1 — Supplementary data to this article can be found online. [file FR-2025-5-0012-Supplementary.zip › 10.48130_forres-0025-0012-Suppl-FigureS3.pdf]

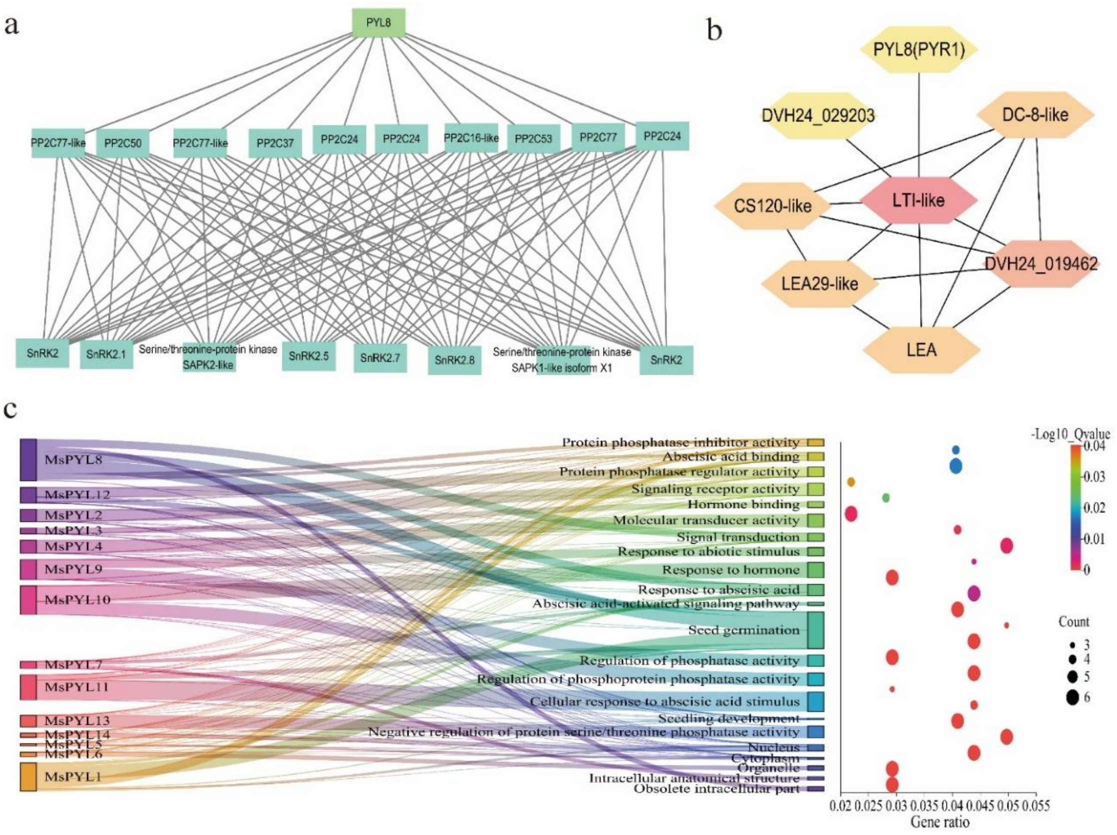

Supplemental Fig. S3 The interaction network and GO enrichment of PYL protein. (a) PYL8 protein interaction network in the ABA signaling pathway. (b) PYL8 interacts with lTI-like. (c) Sankey Diagram with gene ontology (GO) enrichment of PYL proteins.
