# Supplementary material for: Identification of Malus sieversii ABA receptor PYL8 interacting proteome using Y2H-seq
Source: For Res (Fayettev). 2025 Jun 30;5:e012. doi: 10.48130/forres-0025-0012 (PMC12441796; doi:10.48130/forres-0025-0012)
Supplement: Supplementary file 1 — Supplementary data to this article can be found online. [file FR-2025-5-0012-Supplementary.zip › 10.48130_forres-0025-0012-Suppl-FigureS9.pdf]

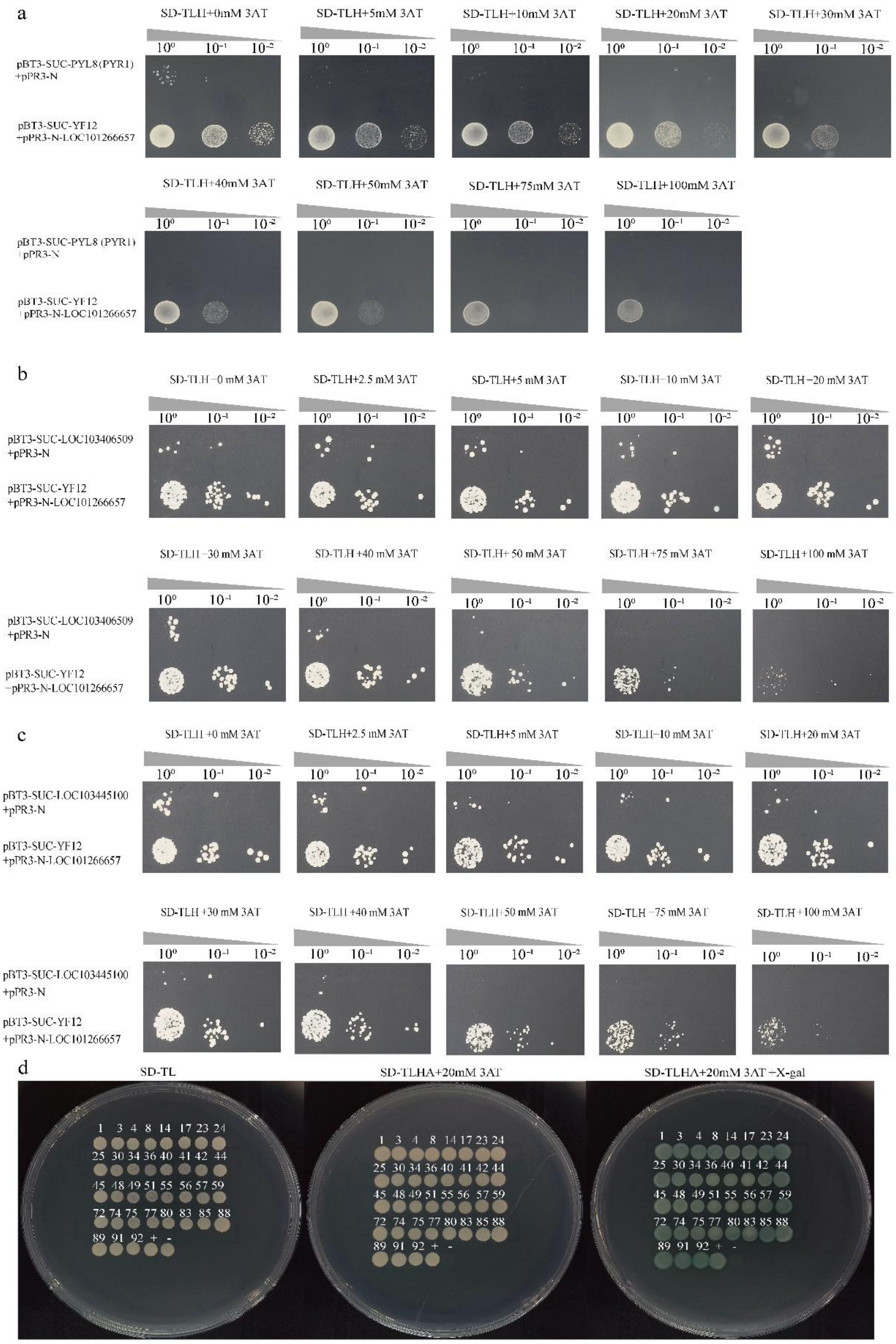

Supplemental Fig. S9 Analysis of self-activating activities. (a) Analysis of MsPYL8 self-activating activities. (b) Analysis of MsLTI(LOC103406509) self-activating activities. (c) Analysis of MsLEA (LOC103445100) self-activating activities. 3AT is a competitive inhibitor of yeast HIS3 protein synthesis, and is used to inhibit leaky expression of *HIS3* genes. SD-TLH is a SD/-Trp-Leu-His-deficient plate. (d) Y2H assay to determine MsPYL8 interactions proteins in yeast rotary validation. (+) Positive control pBT3-SUC-YF12+pPR3-N-LOC101266657, the same as follows. (-) Negative control pBT3-SUC-PYL8(PYR1)+pPR3-N.
