# Supplementary material for: Identification of Malus sieversii ABA receptor PYL8 interacting proteome using Y2H-seq
Source: For Res (Fayettev). 2025 Jun 30;5:e012. doi: 10.48130/forres-0025-0012 (PMC12441796; doi:10.48130/forres-0025-0012)
Supplement: Supplementary file 1 — Supplementary data to this article can be found online. [file FR-2025-5-0012-Supplementary.zip › 10.48130_forres-0025-0012-Suppl-FileS1.docx]

**Supplementary File S1 caption: PYL protein sequences of Malus sieversii, Triticum aestivum, Oryza sativa, Zea mays, Arabidopsis thaliana, and Populus trichocarpa.**

>MsPYL1

MSIGGGDSVEAEYIRRHHRHEPRENQCTSALVRHIKAPVHLVWSLVRRFDQPQKYKPFVSRCTMKGDLGIGSVREVNVKSGLPATTSTERLELLDDDEHILGIRIVGGDHRLRNYSSIITVHREVIEGRPGTLVIESFVVDVPDGNTKDETCYFVEALIRCNLKSLADVSERMAVQDRTEPINQ*

>MsPYL2

MNGNRNGGGGGGGGGGGGFGGIVSDYIRRHHKHDLNDHQCTSTLVRHIKAPVHLVWSLVRRFDQPQKYKPFVSRCVVQGNLEIGSLREVDVKSGLPATTSTERLELLDDDEHILSIKIIGGDHRLRNYSSIISLHPEIIDGRPGTLVIESFVVDIPEGNTKDETCYFVEALIQCNLKSLSDVSERLAVQDQTEPIDRL*

>MsPYL3

MALEKQLKWEGKASAELKGPKAEQIWPLLEDFFGLHEWFPTLTTCHGVEGTSGVAGCVRFCAGFKTPVDPKSDQNQDQEKVNWTKQKLLTIDPANMTYSYSIIDGNVGFNSYISTVQVVPKDAGCTIVWKYEVEPVEGWRLEDLDLFIGTGLQVMASRMEASLQLQVE*

>MsPYL4

MALEKQLKWEGKASAELKVTKTCHGVEGTPGVAGCVWFCAGFKTPVDPKSDQNQDQEKANRTKQKLLRIDPAEMTYSYSIIDGNVGFNSYLSTVQVVPNDAECTIVWKYEVEPVEG*

>MsPYL5

MGRQGKCRAKRPQSRANLASFGDFFGLHKWFPTLTTCLGVEGTQGVAGCLRFCAGVKTPVDHKSDQNQDQEKVNWTKQKLLSIDPAKMTYSYSIIDGNIGFNSYISTVQVVLKDAWCTIVWKYEVEPVEGWRLEDLDLLIGTGLQVTASRMEASLELQVE*

>MsPYL6

MPPNPPKSSVLIHRVHTATSATSNTASSQYQNQRGGGSAALLLNNNSNNNGKKRATVPEAVARHHTHAVGPNQCCSAVTQEIAAPVSTVWSVVRRFDNPQAYKHFVKSCHVIEGGGDVGTLREVQVISGLPANSSTERLEILDDESHVISFSMVGGDHRLSNYRSVTTLHPSSRAGGGTVVVESYVVDVPHGNTNDDTCMFVDTIVRCNLQSLAQIAEDLARCNGQLAAWLGSQEQDHSTDVCMSQHWHN*

>MsPYL7

MSYPTSQNTSPLSESQGMIDTYHVQDLLPNQCGSVLVQTVDAPLTLVWSVLRQFDNPQAYKPFVNSCSIRAGNGGIGSIREVVIKSGLPAKTSTERLDELDDNMHVMHYSVIGGDHRLANYCSTTTVHNVEEENGRKNKTVVIQSYVVDIPAGSSKEDTCLFANTIIGCNLKSLAKVSEKMAATC*

>MsPYL8

MEKSEGSMAEQDAAEETQHHLILPSGLTQNEFDELKSFVNEFHKYHVGPGKCSSLLAQRVHAPPNTVWSVVRRFDQPQAYKHFIRSCNVMEGFEMTVGCTRDVNVISGLPAATSTERLDLLEEDRQVTGFSIIGGEHRLRNYRSVTTVHGFDRDGRISTVVLESYVVDVPEGNSEEDTRLFADTVVKLNLQKLASVTEGMAGDGGR*

>MsPYL9

MDASSAPPYGLTLAEFSELENLIDTYHKFEPSPNTCTSLITQKIEAPAQQVWPLVRSFDSPHRYKHFIKSCNMKGDGGVGSIREVTVVSGLPASTSTERLEILDDEKHIISFRVVGGEHRLNNYRSVTSVNEFVNCKGEEGDNQVYSIVLESYVVDIPEGNTEEDTKMFVDTVIKLNLQKLGVMAMAANLHGSTGGGHDQS*

>MsPYL10

MEQHSDPKWEAKFSTTLKDATVDQIWPLCKDFFYFHKWFPTLAPCYGIHGTNGEPGCIRHCSLFSIPSKSGEKFVSWSKERLIAIDDPDHSLSYKIVESNIGFKSYVSTFRIVPRGDIDGRDGCVIEWSRTVDPGVPSQPCVIRYCATPPTDPVYKTTIRWTKDVLLEIDRIKHRLSYEIIDNNLEFKSYVVVMQLIPVNDQMEQHSDPKWEAKVSATLKDATVDQIWPLFKDFFNFHRWFPTLSTCHGIHGTNGEPGCVRYCSGFSIPSKSGEKSVSWSKERLIAVDDADHSLSYEIVESNLGFNSYVSTFRIVPRGDIDGRDGCVIEWFITVDPVEGWVFGDLVRKYESGLQKMSNRIEDAICKTSGELKGQAAEQLTESSASRVCSDIAPLLRLILPMRRPSSGPKRFYLRLIRSN*

>MsPYL11

MPLVPVNGEDGSTGCRVEWSFVTISLIMQEPAESRKWERKTTAVLKASAAEKVWPLLADFCNLHKIFPKLATCYQVEGVSGQPGLIRYCETPPTNPADESTVKWVKEKLLMIDPIKRCISYEIIENNMGFHSYVATMQVVPTNDIDQDGVYGCKIDWSVVCDPVDGWRSENLQEFLESNLQLVAKTMEHALLST*

>MsPYL12

MEEEEDQKQSKWQGNASAELKRTAAEQAWPALADFCNLHKWFPNLATCRQVEGIPGQPGVIRYCASAPVDNDESSIKWAKEKLLTIDPIQRCLSYEVTESNLGFKSYVSTMQVVAMDGGGGCKIEWSFVCDPIEGLGLDEFLSYLDSSLQLMGKAIMEHAPPSTAN*

>MsPYL13

MDAGHAPPYGLTLAEFSELEGLIDAYHKFEPSPNTCTSLITQKIEAPAQQIWPLVRNFDNPKKYKHFIKSCNMKGDGGVGSIREVTVVSGLPASTSTERLEILDDEKHIISFRVMGGEHRLNNYRSVTSVNEFVNCDGEEGNNQVYSIVLESYVVDIPGGNTGEDTKMFVDTVIKLNLQKLGVVAMAANVVHGNTGGGHDQS*

>MsPYL14

MGENQSPLKWEGKTSRELKGHAAEQVWPLWADFCNFHKWLPVLDACYLVDGVPSQPGVTRSEALKLRNSEALKHPGSRRIKKAFFVLHSSFFQDQAPTALEELPPIQDQAPTALEESTIVHHPFIQDQSPNGPLDQQRRQIHTHPTIRSNPLSKIKPQRPLWIDNINESAYPLFIKPKPRRPLKKAFIVHHYSSRSSPKALEDPFKPKSSRRSVRHCSSSSSPKALGNPFITVLQDQAQKPLKIRQSPFFKIKPKSPLKIRSSSFIQDQASTALGSIAHPTNQHLTEIESEDQI*

>AtPYL9

MMDGVEGGTAMYGGLETVQYVRTHHQHLCRENQCTSALVKHIKAPLHLVWSLVRRFDQPQKYKPFVSRCTVIGDPEIGSLREVNVKSGLPATTSTERLELLDDEEHILGIKIIGGDHRLKNYSSILTVHPEIIEGRAGTMVIESFVVDVPQGNTKDETCYFVEALIRCNLKSLADVSERLASQDITQ*

>AtPYL3

MNLAPIHDPSSSSTTTTSSSTPYGLTKDEFSTLDSIIRTHHTFPRSPNTCTSLIAHRVDAPAHAIWRFVRDFANPNKYKHFIKSCTIRVNGNGIKEIKVGTIREVSVVSGLPASTSVEILEVLDEEKRILSFRVLGGEHRLNNYRSVTSVNEFVVLEKDKKKRVYSVVLESYIVDIPQGNTEEDTRMFVDTVVKSNLQNLAVISTASPT*

>AtPYL2

MSSSPAVKGLTDEEQKTLEPVIKTYHQFEPDPTTCTSLITQRIHAPASVVWPLIRRFDNPERYKHFVKRCRLISGDGDVGSVREVTVISGLPASTSTERLEFVDDDHRVLSFRVVGGEHRLKNYKSVTSVNEFLNQDSGKVYTVVLESYTVDIPEGNTEEDTKMFVDTVVKLNLQKLGVAATSAPMHDDE*

>AtPYL4

MLAVHRPSSAVSDGDSVQIPMMIASFQKRFPSLSRDSTAARFHTHEVGPNQCCSAVIQEISAPISTVWSVVRRFDNPQAYKHFLKSCSVIGGDGDNVGSLRQVHVVSGLPAASSTERLDILDDERHVISFSVVGGDHRLSNYRSVTTLHPSPISGTVVVESYVVDVPPGNTKEETCDFVDVIVRCNLQSLAKIAENTAAESKKKMSL*

>AtPYL6

MPTSIQFQRSSTAAEAANATVRNYPHHHQKQVQKVSLTRGMADVPEHVELSHTHVVGPSQCFSVVVQDVEAPVSTVWSILSRFEHPQAYKHFVKSCHVVIGDGREVGSVREVRVVSGLPAAFSLERLEIMDDDRHVISFSVVGGDHRLMNYKSVTTVHESEEDSDGKKRTRVVESYVVDVPAGNDKEETCSFADTIVRCNLQSLAKLAENTSKFS*

>AtPYL7

MEMIGGDDTDTEMYGALVTAQSLRLRHLHHCRENQCTSVLVKYIQAPVHLVWSLVRRFDQPQKYKPFISRCTVNGDPEIGCLREVNVKSGLPATTSTERLEQLDDEEHILGINIIGGDHRLKNYSSILTVHPEMIDGRSGTMVMESFVVDVPQGNTKDDTCYFVESLIKCNLKSLACVSERLAAQDITNSIATFCNASNGYREKNHTETNL*

>AtPYR1

MPSELTPEERSELKNSIAEFHTYQLDPGSCSSLHAQRIHAPPELVWSIVRRFDKPQTYKHFIKSCSVEQNFEMRVGCTRDVIVISGLPANTSTERLDILDDERRVTGFSIIGGEHRLTNYKSVTTVHRFEKENRIWTVVLESYVVDMPEGNSEDDTRMFADTVVKLNLQKLATVAEAMARNSGDGSGSQVT*

>AtPYL13

MESSKQKRCRSSVVETIEAPLPLVWSILRSFDKPQAYQRFVKSCTMRSGGGGGKGGEGKGSVRDVTLVSGFPADFSTERLEELDDESHVMVVSIIGGNHRLVNYKSKTKVVASPEDMAKKTVVVESYVVDVPEGTSEEDTIFFVDNIIRYNLTSLAKLTKKMMK*

>AtPYL10

MNGDETKKVESEYIKKHHRHELVESQCSSTLVKHIKAPLHLVWSIVRRFDEPQKYKPFISRCVVQGKKLEVGSVREVDLKSGLPATKSTEVLEILDDNEHILGIRIVGGDHRLKNYSSTISLHSETIDGKTGTLAIESFVVDVPEGNTKEETCFFVEALIQCNLNSLADVTERLQAESMEKKI*

>AtPYL5

MRSPVQLQHGSDATNGFHTLQPHDQTDGPIKRVCLTRGMHVPEHVAMHHTHDVGPDQCCSSVVQMIHAPPESVWALVRRFDNPKVYKNFIRQCRIVQGDGLHVGDLREVMVVSGLPAVSSTERLEILDEERHVISFSVVGGDHRLKNYRSVTTLHASDDEGTVVVESYIVDVPPGNTEEETLSFVDTIVRCNLQSLARSTNRQ*

>AtPYL11

METSQKYHTCGSTLVQTIDAPLSLVWSILRRFDNPQAYKQFVKTCNLSSGDGGEGSVREVTVVSGLPAEFSRERLDELDDESHVMMISIIGGDHRLVNYRSKTMAFVAADTEEKTVVVESYVVDVPEGNSEEETTSFADTIVGFNLKSLAKLSERVAHLKL*

>AtPYL12

MKTSQEQHVCGSTVVQTINAPLPLVWSILRRFDNPKTFKHFVKTCKLRSGDGGEGSVREVTVVSDLPASFSLERLDELDDESHVMVISIIGGDHRLVNYQSKTTVFVAAEEEKTVVVESYVVDVPEGNTEEETTLFADTIVGCNLRSLAKLSEKMMELT*

>AtPYL1

MANSESSSSPVNEEENSQRISTLHHQTMPSDLTQDEFTQLSQSIAEFHTYQLGNGRCSSLLAQRIHAPPETVWSVVRRFDRPQIYKHFIKSCNVSEDFEMRVGCTRDVNVISGLPANTSRERLDLLDDDRRVTGFSITGGEHRLRNYKSVTTVHRFEKEEEEERIWTVVLESYVVDVPEGNSEEDTRLFADTVIRLNLQKLASITEAMNRNNNNNNSSQVR*

>AtPYL8

MEANGIENLTNPNQEREFIRRHHKHELVDNQCSSTLVKHINAPVHIVWSLVRRFDQPQKYKPFISRCVVKGNMEIGTVREVDVKSGLPATRSTERLELLDDNEHILSIRIVGGDHRLKNYSSIISLHPETIEGRIGTLVIESFVVDVPEGNTKDETCYFVEALIKCNLKSLADISERLAVQDTTESRV*

>ZmPYL1

MDQQGAGGDVEVPAGLGLTAAEYEQLRPTVDAHHRYAVGEGQCSSLLAQRIHAPPAAVWAIVRRFDCPQVYKHFIRSCAVRPDPDAGDALRPGRLREVCVISGLPASTSTERLDHLDDAARVFGFSITGGEHRLRNYRSVTTVSELAGPGICTVVLESYAVDVPDGNTEDDTRLFADTVIRLNLQKLKSVAEASTSSSAPPPPSE

>ZmPYL2

MDQQGAGGDAEVPAGLGLTAAEYEQLRSTVDAHHRYAVGEGQCSSLLAQRIHAPPEAVWAVVRRFDCPQVYKHFIRSCALRPDPEAGDALCPGRLREVSVISGLPASTSTERLDLLDDAARVFGFSITGGEHRLRNYRSVTTVSELADPAICTVVLESYVVDVPDGNTEDDTRLFADTVIRLNLQKLKSVAEANAAEAAATTNSVLLPRPAE

>ZmPYL3

MEPHMESALRQGLSEAEQRELEGVVRAHHTFPGRAPGTCTSLVTQRVDAPLAAVWPIVRGFGSPQRYKHFIKSCDLKAGDGATVGSVREVTVVSGLPASTSTERLEILDDHRHILSFRVVGGDHRLRNYRSVTSVTEFQPGPYCVVLESYVVDVPDGNTEEDTRMFTDTVVKLNLQKLAAIATSSSAN

>ZmPYL4

MPYTAPRPSPQQHSRVLSGGGAKAASHGASCAAVPAEVARHHEHAARAGQCCSAVVQAIAAPVGAVWSVVRRFDRPQAYKHFIRSCRLVGGGDVAVGSVREVRVVSGLPATSSRERLEILDDERRVLSFRVVGGEHRLANYRSVTTVHEAGAGAGTGTVVVESYVVDVPHGNTADETRVFVDTIVRCNLQSLARTAERLA

>ZmPYL5

MPCLQASSPGSMPYQHHGRGVGCAAEAGAAVGASAGTGTRCGAHDGEVPAEAARHHEHAAPGPGRCCSAVVQRVAAPAEAVWSVVRRFDQPQAYKRFVRSCALLAGDGGVGTLREVRVVSGLPAASSRERLEVLDDESHVLSFRVVGGEHRLQNYLSVTTVHPSPAAPDAATVVVESYVVDVPPGNTPEDTRVFVDTIVKCNLQSLATTAEKLALAAV

>ZmPYL6

MPCIQASSPGGMPHQHGRGRVLGGGVGCAAEVAAAVAASAGGMRCGAHDGEVPAEAARHHEHAAAGPGRCCSAVVQHVAAPAAAVWSVVRRFDQPQVYKRFVRSCALLAGDGGVGTLREVRVVSGLPAASSRERLEVLDDESHVLSFRVVGGEHRLRNYLSVTTVHPSPAAPDAATVVVESYVVDVPPGNTPEDTRVFVDTIVKCNLQSLATTAEKLAAV

>ZmPYL7

MPYAATRTSPQQHSRVASNGRAVAACAGHAGVPDEVARHHEHAVAAGQCCSVMVQSIAAPADAVWSLVRRFDQPQGYKRFIRSCHLVDGDGVEVGSVRELLVVSGLPAENSRERLEIRDDERRVISFRILGGDHRLANYRSVTTVHEAASEGGPLTMVVESYVVDVPPGNTVEETRIFVDTIVRCNLQSLEDTVIRQQAMAAPAAPHNDHNHS

>ZmPYL8

MVGLVGGSTARAEHVVANAGGEAEYVRRMHRHAPTEHQCTSTLVKHIKAPVHLVWELVRRFDQPQRYKPFVRNCVVRGDQLEVGSLRDVNVKTGLPATTSTERLEQLDDDLHILGVKFVGGDHRLQVRAAAARLLRPCKNALFHAPSLLVSVLQIIFGGMLALLPFLFFLN

>ZmPYL9

MVGLVGGSTARAEHVVANAGGETEYVRRLHRHAPAEHQCTSTLVKHIKAPVHLVWELVRSFDQPQRYKPFVRNCVVRGDQLEVGSLRDVNVKTGLPATTSTERLEQLDDDLHILGVKFVGGDHRLQNYSSIITVHPESIDGRPGTLVIESFVVDVPDGNTKDETCYFVEAVIKCNLKSLAEVSEQLAVESPTSPIDQ

>ZmPYL10

MVMVEMDGGVGGGGGGGQTPAPRRWRLADERCDLRAMETDYVRRFHRHEPREHQCSSAVAKHIKAPVHLVWSLVRRFDQPQLFKPFVSRCEMKGNIEIGSVREVNVKSGLPATRSTERLELLDDNEHILSVRFVGGDHRLQNYSSILTVHPEVIDGRPGTLVIESFVVDVPDGNTKDETCYFVEALLKCNLKSLAEVSERQVVKDQTEPLDR

>ZmPYL11

MVVEMDGGVGVAAAGGGGAQTPAPPPPRRWRLADERCDLRAMETDYVRRFHRHEPRDHQCSSAVAKHIKAPVHLVWSLVRRFDQPQLFKPFVSRCEMKGNIEIGSVREVNVKSGLPATRSTERLELLDDDERILSVRFVGGDHRLQVCSVLHLSIFCAAHARYFAHHLKCVLEFLCQMHLDVLPCDDAILE

>ZmPYL12

MVGLVGGSTARAEHVVANAGGEAEYVRRMHRHAPTEHQCTSTLVKHIKAPVHLVWELVRRFDQPQRYKPFVRNCVVRGDQLEVGSLRDVNVNPGLPATTSTERLEQLDDDLHILGVKFVGGDHRLQLAGVVAVEVTGGPDVPFHPGREFKQIVMNMLDCFRLTVEYGVRSSQNLGDEQPQACEIKAQLVRTAEAARQLALMLEVERPSYQGRNSVYQSSSKMMSAISVAHLGCKDMDAVDVGVVGMVDSQSLSSALEKLYFWERKLYAEVK

>ZmPYL13

MRERNSSIDQEHQRGSSSRSTMPFAASRTSQQQHSRVATNGRAVAVCAGHAGVPDEVARHHEHAVAAGQCCAAMVQSIAAPVDAVWSLVRRFDQPQRYKRFIRSCHLVDGDGAEVGSVRELLLVSGLPAESSRERLEIRDDERRVISFRVLGGDHRLANYRSVTTVHEAAPSQDGRPLTMVVESYVVDVPPGNTVEETRIFVDTIVRCNLQSLEGTVIRQLEIAAMPHDDNQN

>OsPYL1

MEQQEEVPPPPAGLGLTAEEYAQVRATVEAHHRYAVGPGQCSSLLAQRIHAPPAAVWAVVRRFDCPQVYKHFIRSCVLRPDPHHDDNGNDLRPGRLREVSVISGLPASTSTERLDLLDDAHRVFGFTITGGEHRLRNYRSVTTVSQLDEICTLVLESYIVDVPDGNTEDDTRLFADTVIRLNLQKLKSVSEANANAAAAAAAPPPPPPAAAE*

>OsPYL2

MEAHVERALREGLTEEERAALEPAVMAHHTFPPSTTTATTAAATCTSLVTQRVAAPVRAVWPIVRSFGNPQRYKHFVRTCALAAGDGASVGSVREVTVVSGLPASTSTERLEMLDDDRHIISFRVVGGQHRLRNYRSVTSVTEFQPPAAGPAPAPPYCVVVESYVVDVPDGNTAEDTRMFTDTVVKLNLQKLAAVAEDSSSASRRRD*

>OsPYL3

MEPHMERALREAVASEAERRELEGVVRAHHTFPAAERAAGPGRRPTCTSLVAQRVDAPLAAVWPIVRGFANPQRYKHFIKSCELAAGDGATVGSVREVAVVSGLPASTSTERLEILDDDRHVLSFRVVGGDHRLRNYRSVTSVTEFSSPSSPPSPPRPYCVVVESYVVDVPEGNTEEDTRMFTDTVVKLNLQKLAAVATSSSPPAAGNHH*

>OsPYL4

MPYAAVRPSPPPQLSRPIGSGAGGGKACPAVPCEVARYHEHAVGAGQCCSTVVQAIAAPADAVWSVVRRFDRPQAYKKFIKSCRLVDGDGGEVGSVREVRVVSGLPATSSRERLEVLDDDRRVLSFRIVGGEHRLANYRSVTTVHEAAAPAMAVVVESYVVDVPPGNTWEETRVFVDTIVRCNLQSLARTVERLAPEAPRANGSIDHA*

>OsPYL5

MMPYTAPRPSPPQHSRIGGCGGGGVLKAAGAAGHAASCVAVPAEVARHHEHAAGVGQCCSAVVQAIAAPVDAVWSVVRRFDRPQAYKHFIRSCRLLDGDGDGGAVAVGSVREVRVVSGLPATSSRERLEILDDERRVLSFRVVGGEHRLSNYRSVTTVHETAAGAAAAVVVESYVVDVPHGNTADETRMFVDTIVRCNLQSLARTAEQLALAAPRAA*

>OsPYL6

MPCIPASSPGIPHQHQHQHHRALAGVGMAVGCAAEAAVAAAGVAGTRCGAHDGEVPMEVARHHEHAEPGSGRCCSAVVQHVAAPAPAVWSVVRRFDQPQAYKRFVRSCALLAGDGGVGTLREVRVVSGLPAASSRERLEILDDESHVLSFRVVGGEHRLKNYLSVTTVHPSPSAPTAATVVVESYVVDVPPGNTPEDTRVFVDTIVKCNLQSLANTAEKLAAGARAAGS*

>OsPYL7

MNSGAGGAGGAAVGRMPAGSLQWAQWRLADERCELREEEMEYMRRFHRHEIGSNQCNSFIAKHVRAPLQNVWSLVRRFDQPQIYKPFVRKCVMRGNVETGSVREIIVQSGLPATRSIERLEFLDDNEYILRVKFIGGDHMLKKCGP

>OsPYL8

MNGAGGAGGAAAGKLPMVSHRQVQWRLADERCELREEEMEYIRQFHRHEPSSNQCTSFVAKHIKAPLQTVWSLVRRFDQPQLFKPFVRKCVMRENIIATGCVREVNVQSGLPATRSTERLELLDDNEHILKVKFIGGDHMLKNYSSILTIHSEVIDGQLGTLVVESFVVDIPEGNTKDDICYFIENILRCNLMTLADVSEERLANP*

>OsPYL9

MNGVGGAGGAAAGKLPMVSHRRVQWRLADERCELREEEMEYIRRFHRHEPSSNQCTSFAAKHIKAPLHTVWSLVRRFDQPQLFKPFVRNCVMRENIIATGCIREVNVQSGLPATRSTERLELLDDNEHILKVKFIGGDHMLKNYSSILTIHSEVIDGQLGTLVVESFIVDVLEGNTKDDISYFIENVLRCNLRTLADVSEERLANP*

>OsPYL10

MVEVGGGAAEAAAGRRWRLADERCDLRAAETEYVRRFHRHEPRDHQCSSAVAKHIKAPVHLVWSLVRRFDQPQLFKPFVSRCEMKGNIEIGSVREVNVKSGLPATRSTERLELLDDNEHILSVRFVGGDHRLKNYSSILTVHPEVIDGRPGTLVIESFVVDVPEGNTKDETCYFVEALLKCNLKSLAEVSERLVVKDQTEPLDR*

>OsPYL11

MVGLVGGGGWRVGDDAAGGGGGGAVAAGAAAAAEAEHMRRLHSHAPGEHQCSSALVKHIKAPVHLVWSLVRSFDQPQRYKPFVSRCVVRGGDLEIGSVREVNVKTGLPATTSTERLELLDDDEHILSVKFVGGDHRLRNYSSIITVHPESIDGRPGTLVIESFVVDVPDGNTKDETCYFVEAVIKCNLTSLAEVSERLAVQSPTSPLEQ*

>OsPYL12

MRGSTSLAVGCVREVDFKSGFPAKSSVERLEILDDKEHVFGVRIIGGDHRLKNYSSVLTAKPEVIDGEPATLVSESFVVDVPEGNTADETRHFVEFLIRCNLRSLAMVSQRLLLAQGDLAEPPAQ*

>OsPYL13

MNGCTGGAGGVAAGRLPAVSLQQAQWKLVDERCELREEEMEYVRWFHRYELVATGATPSLPNTSGCPSKLGLPSTRRIERLGFPDDNDHTLRVKFIGGDHMLKDYSSTLIIHLEVIDGQLVTLVIESFVVDILEGNTKDEISYFIENLLKFNLRTLRV*

>TaPYL1

MEKVTQESEQQAWQGAVEALLPSTPAAAAWPHLASFCALHRYLSGVDVCERVAGEDGHPGCVRYVASRAAPAPGEEDQDQHEAAPAAIATWAREELLELDDAARRLSYAVVGSNMGFGRYVATMMVVEEETEAAGCKLVWEFECEPVQGWSRDGLVGYLETTVKSMAARIVEAAAD*

>TaPYL2

MVGLVGGGARAWRLSDEAANGAGGGGAATEADYMRRLHGHAPGENQCTSALVKHIKAPVHLVWSLVRSFDQPQRYKPFVSRCVVRGGDLEIGSVREVNVKTGLPATTSTERLEQLDDDEHILSVKFVGGDHRLRNYSSIITVHPQSIDGRPGTLVIESFVVDVPDGNTKDETCYFVEAVIKCNLTSLAEVSERLAVQSPTSPLEQ*

>TaPYL3

MEQQPVAAAEPEVPAGLGLTAAEYAQLLPTVEAYHRYAVGPGQCSSLVAQRIEAPPAAVWAIVRRFDCPQVYKHFIRSCALRPDPEAGDELRPGRLREVSVISGLPASTSTERLDLLDDARRAFGFTITGGEHRLRNYRSVTTVSELSPAAPAEICTVVLESYVVDVPDGNSEEDTRLFADTVVRLNLQKLKSVAEANAAAAAATPAPPAE*

>TaPYL4

MPYTASRPSAPQRARVAAGGGGWKAAAHAASCGAVPGEVARHHEHAAGAGQCCSAVVQAIEAPVGAVWAVVRRFDRPQAYKHFIRSCRVVDGDGGAVGSVREVRVVSGLPATSSRERLEILDDERRVLSFRVVGGEHRLSNYRSVTTVHEAASAGAVVVESYVVDVPPGNTADETRTFVDTIVRCNLQSLARTAQQLALAA*

>TaPYL5

MEKATQESEQEAWQGAVEALLPSTPAAAAWPHLASFCALHRYLSGVDVCKRVAGEDGHPGCVRYVASRAAPAPGEEDQDQHEAAPAAIATWAREELLELDDAARRLSYAVVGSNMGFGRYVATMMVVEEETEAAGCKLVWEFECEPVQGWSRDGLVGYLETTVKGMAARIVEAAAD*

>TaPYL6

MVGLLGGGARAWRLSDEAANGAVGGGAATEADYMRRLHGHAPGENQCTSALVKHIKAPVHLVWSLVRSFDQPQRYKPFVSRCVVRGGDLEIGSVREVNVKTGLPATTSTERLEQLDDDEHILSVKFVGGDHRLRNYSSIITVHPQSIDGRPGTLVIESFVVDVPDGNTKDETCYFVEAVIKCNLTSLAEVSERLAVQSPTSPLEQ*

>TaPYL7

MEQQPVAAAAAAEPEVPAGLGLTAAEYAQLLPTVEAYHRYAVGPGQCSSLVAQRIEAPPAAVWAIVRRFDCPQVYKHFIRSCALRPDPEAGDELRPGRLREVSVISGLPASTSTERLDLLDDARRAFGFTITGGEHRLRNYRSVTTVSELSPAAPAEICTVVLESYVVDVPDGNSEEDTRLFADTVVRLNLQKLKSVAEANAAAAATTAPPAE*

>TaPYL8

MPYTASRPSAPQRARVAAVGAGWKAAAHAASCGAVPGEVARHHEHAAGTGQCCSAVVQAIEAPVGAVWAVVRRFDRPQAYKHFIRSCRLVDGDGGAVGSVREVRVVSGLPATSSRERLEILDDERRVLSFRVVGGEHRLSNYRSVTTVHEAASAGAVVVESYVVDVPPGNTADETRTFVDTIVRCNLQSLARTA*

>TaPYL9

MVGLVGGGARAWRLSDEAANGAGGGGVATEADYMRRLHGHAPGENQCTSALVKHIKAPVHLVWSLVRSFDQPQRYKPFVSRCVVRGGDLEIGSVREVNVKTGLPATTSTERLEQLDDDEHILSVKFVGGDHRLRNYSSIITVHPQSIDGRPGTLVIESFVVDVPDGNTKDETCYFVEAVIKCNLTSLAEVSERLAVQSPTSPLEQ*

>TaPYL10

MEKATQESEKQAWQGVVEALLPSTPAAAAWPHLASFCALHRYLSGVDVCERVAGEDGHPGCVRYVASRAAPAPGEEDQDQQEAAPAAIATWAREELLELDDAARRLSYAVVGSNMGFGRYVATMMVVEEETEAAGCKLVWEFECEPVQGWSRDGLVGYLETTVKGMAARIVEAAAD*

>TaPYL11

MEQQPVAAAATEPEVPAGLGLTAAEYAQLLPTVEAYHRYAVGPGQCSSLVAQRIEAPPAAVWAIVRRFDCPQVYKHFIRSCALRPDPEAGDELRPGRLREVSVISGLPASTSTERLDLLDDARRAFGFTITGGEHRLRNYRSVTTVSELSPAAPAEICTVVLESYVVDVPDGNSEEDTRLFADTVVRLNLQKLKSVAEANAAAAAATPAPPAE*

>TaPYL12

MPYTASRPSAPQRARVAAVGAGWKAAAHAASCGAVPGEVARHHEHAAGAGQCCSAVVQAIEAPVGAVWAVVRRFDRPQAYKHFIRSCRVVDGDGGAVGSVREVRVVSGLPATSSRERLEILDDERRVLSFRVVGGEHRLSNYRSVTTVHEAAPAGAVVVESYVVDVPPGNTADETRTFVDTIVRCNLQSLARTAQQLAVPA*

>TaPYL13

MPRRLAATGSALLPGEPQVLRTIYIVRLAPPTFNLLPNQAKRSSAAARSAPIPPNKQLAGEATRSALEPMPTPYSAAALQQHHRLVSSSGGLATAAAAGAHRCGEHDGTVPPEVARHHEHAAPGGRCCCSAVVQRVAAPAADVWAVVRRFDQPQAYKSFVRSCALLDGDGGVGTLREVRVVSGLPAASSRERLEILDDERHVLSFSVVGGEHRLRNYRSVTTVHPAPGESASATLVVESYVVDVPPGNTPEDTRVFVDTIVKCNLQSLARTAEKLAGRGPAYGALP*

>TaPYL14

MPTPYSAAALQQHHRLVSSSGGLAVAAATGAHRCGEHDGTVPPEVARHHEHAAPGGRCCCSAVVQRVAAPAADVWAVVRRFDQPQAYKSFVRSCALLDGDGGVGTLREVRVVSGLPAASSRERLEILDDERHVLSFSVVGGEHRLRNYRSVTTVHPAPGGSASATLVVESYVVDVPPGNTPEDTRVFVDTIVKCNLQSLARTAEKLAGRGAAYGALP*

>TaPYL15

MPTPYSAAALQQHQRLVSSSGGLAATGAHRCGEHDGTVPPEVARHHEHAAPGGRCCCSAVVQRVAAPAADVWAVVRRFDQPQAYKSFVRSCALLDGDGGVGTLREVRVVSGLPAASSRERLEILDDERHVLSFSVVGGEHRLRNYRSVTTVHPAPGESASATLVVESYVVDVPPGNTPEDTRVFVDTIVKCNLQSLARTAEKLAGRGAAYGALP*

>TaPYL16

MESALRQGLTEPERREVEGVVEEHHTFPGRASGTCTSLVTQRVQAPLAAVWDIVRGFANPQRYKHFIKSCALAAGDGATVGSVREVTVVSGLPASTSTERLEILDDDRHILSFRVVGGEHRLRNYRSVTSVTEFTDQPSGPPYCVVVESYVVDVPEGNTEEDTRMFTDTVVKLNLQKLAAIATTTSSSSPPPSDEQS*

>TaPYL17

MAPHGHGPDSETAAPEWRGTVRAAAAGPTPDQAWALLGDFCSLDKWVSLVQTCRRLEGDDGRPGCVRYCAGPVNMAAPGEAVGWSKERLLEVDAAGRSYSYEVVETNKGFGRYRATIGVEPDPAGCAVRWSFEADPVEGWTLEGFVGFLEKLAHGVAKRLEEEIMANVDGDPAALRVF*

>TaPYL18

MDVPTAGGAAAAAPQRRAWEWEGRVVSPVPAATADEAWALLSDFLAFHKWHPRVAVCRLASASPSGAAAAPGCVRYCEGTPPGDGAPADWAHETLLEHDQARRFFRYEMNDNNMGFGAFFAAFRVVPAAHAAGGCELRWEFECEPVRGTPKEALEARLQAGLDGMAARVHEHLMSARAAVAAAAPAPAVAAGLEAADELKLDNSIAA*

>TaPYL19

MEVGEAGAQQGRALAAAAPEQWRGAVEAALPGTPAGAAWAHVASFFSAHRYLPGIDVCERVVTGAGCEEDDDGRLPIAPGCVRHVASSAAGLWAREELLEADHAARRLRYAVVDSNMGFGRYVATLRVLGLDGGEGGCMISWAFECDAVEGEGWSEAALVARLGASVKGMAERVQQLAAVPQ*

>TaPYL20

MPYAAARPSPQQHSRISSGCKALVAHGAAVPGEVARYHEHAAGAGQCCSAVVQAIAAPVEAVWSVVRRFDRPQAYKRFIKSCRMVDGDGGAVGSVREVRVVSGLPGTSSRERLEILDDERRVLSFRIVGGEHRLANYRSVTTVSEVASTVAGAPRVTLVVESYVVDVPPGNTSDETRLFVDTIVRCNLQSLARTAEQLALAVPHVN*

>TaPYL21

MEIHKEGAQQGRAPEAAEAPEQWRGAVEAPLPGTPAGAAWAHVASFFSAHLYLPGIDVCERVSGASSEEDEDGRLVITPGCVRHVASSAAGLWAREELLEADHAARRLRYAVVDSNMGFGRYVATLRVLDLDGGEGGCMISWAFECDAVKSEGWSEAALVARLGASVKGMAERVQQLAAVAQ*

>TaPYL22

MDVPTAGQAAAVPQRKALEWEGRVVSPVPTATADEAWALLSDFLAFHKWHPRVAVCRLASSGAAAPGCVRYCEGTPPADGTPADWAHETLLEHDQARRFFRYEMNDNNMGFGAFFAAFRVVPAAAGGCLLRWEFECEPVRGTPKEALEARLQAGLDGMAARVHEHLMSARGAVAAAAPPT

AVTAGLEAADELKLDSSIAA*

>TaPYL23

MAPHDPESETAAPEWRGTVRAAAAGPTPDQAWALLRDFCSLNKWVSLVETCRRLEGDDGRPGCVRYCAGPVNMAAPGEAVGWSKERLLEVDAAGRSYSYEVVETNKGFGRYRATIGVEPDPAGCAVRWSFEADPVKGWTLEGFVGFLEKLAHGVAKRLEEEIMVNVDGDPALPIF*

>TaPYL24

MPYAAARPSLQQHSRISSGCKALVAHGAAVPGEVALYHEHAAGAGQCCSAVVQAIAAPVEAVWSVVRRFDRPQAYKRFIKSCRVVDGDGGAVGSVREVRVVSGLPGTSSRERLEILDDERRVLSFRIVGGEHRLANYRSVTTVNEVASTVAAGAPRVTLVVESYVVDVPPGNTSDETRLFVDTIVRCNLQSLARTAEQLALAVPHVN*

>TaPYL25

MEIDKEGAQQGRALEAAEAPEQWRGAVEAPLPGTPVGAAWAHVASFFSAHIYLPGIDVCERVTGASSEDDEDGRLVITPGCVRHVASSAAGLWAREELLESDHAARRLRYAVVDSNMGFGRYVATLRVLDLDDGGEGGCVISWAFECDAVKAEGWSEAALVARLGASVKGMAERVQQLAAVAQ*

>TaPYL26

MDVPTAGQAAAAPQRKAWEWEGRVVSPVPAATADEAWALLSDFLAFHKWHPRVAVCRLASSSGAAAPGCVRYCEGTPPADGTPADWAHETLLEHDQARRFFRYEMNDNNMGFGAFFAAFRVVPAAAGGCVLRWEFECEPVRGTPKEALEARLQAGLDGMAARVHEHLMSARAAVAVAAATAPTTTVTAGLEAADELKLDSSIAA*

>TaPYL27

MEPHMESALRQGLTEPERREVEGVVEEHHTFPGRASGTCTSLVTQRVQAPLAAVWDIVRGFANPQRYKHFIKSCALAAGDGATVGSVREVTVVSGLPASTSTERLEILDDDRHILSFRVVGGEHRLRNYRSVTSVTEFADEPSGPSYCVVVESYVVDVPEGNTEEDTRMFTDTVVKLNLQKLAAIATTTTSSSPPPSDEQS*

>TaPYL28

MAPHGPDGETAPEWRGTVRAAAAGPTPDQAWALLGDFCSLDKWVSLVQTCRRLEGDDGQPGCVRYCAGPVNMAAPGEAVGWSKERLLEVDAAGRSYSYEVVETNKGFGRYRATIGVQPDPAGCAVRWSFEADPVKGWTLEGFVGFLEKLAHGVAKRLEEEIMVNVDGDPAALRVF*

>TaPYL29

MPYAAARPSPQQHSRISAGCKALVAHGAAVPGEVARYHEHAAGAGQCCSAVVQAIAAPVEAVWSVVRRFDRPQAYKRFIKSCRLVDGDGGAVGSVREVRVVSGLPGTSSRERLEILDDERRVLSFRIVGGEHRLANYRSVTTVSEVASTVAGAPRVTLVVESYVVDVPPGNTSDETRMFVDTIVRCNLQSLARTAEQLALAVPHVN*

>TaPYL30

MRCREHDCEVPAEVARHHEHAEPGSGQCCSAVVQHVAAPAAAVWSVVRRFDQPQAYKRFVRSCALVAGDGGVGTLREVHVVSGLPAASSRERLEILDDESHVLSFRVVGGEHRLKNYLSVTTVHPSPAAPSSATVVVESYVVDVPAGNTTEDTRVFIDTIVKCNLQSLAKTAEKVAAVS*

>TaPYL31

MPCIPVSSPSIQHHNHNHHHRVLAGVGVGMGCGAEAVVAAAGTAGMRCREHDCEVPAEVARHHEHAEPGSGQCCSAVVQHVAAPAAAVWSVVRRFDQPQAYKRFVRSCALVAGDGGVGTLREVHVVSGLPAASSRERLEILDDESHVLSFRVVGGEHRLKNYLSVTTVHPSPAAPSSATVVVESYVVDVPAGNTIEDTRVFIDTIVKCNLQSLAKTAEKLAAVS*

>TaPYL32

MPCIPASSPSIQHHNHNHHHRVLAGVGVGMGCGAEAVVAAAGTAGMRCREHDCEVPAEVARHHEHAEPGSGQCCSAVVQHVAAPAAAVWSVVRRFDQPQAYKRFVRSCALVAGDGGVGTLREVHVVSGLPAASSRERLEILDDESHVLSFRVVGGEHRLKNYLSVTTVHPSPAAPSSATVVVESYVVDVPAGNTIEDTRVFIDTIVKCNLQSLAKTAEKVAAVS*

>TaPYL33

MDGGSSGVGADGIWRPWDEHTVLRPEEMEYVRRFHQHVPGANQCTSFIAKHIKAPLQTVWSVVRRFDKPQVYKRFVENCVMQGNIEPGCVREVTLKSGLPGKWSIERLELLDDNEHILSVMFIDGDHPLKNYSSILTVHHEVADGHPGALVIESFVVDIPKENTENEIFYLVGNFLKFNH

KLLADVSEGQIDRRALN*

>TaPYL34

MEAHMERALREGVTEAERAALEGTVRAHHTFPGRAPGGTCTSLVAQRVAAPVRAVWPIVRSFGNPQRYKHFVRTCALAAGDGASVGSVREVTVVSGLPASTSTERLEILDDDRHILSFSVVGGDHRLRNYRSVTSVTEFQPGPYCVVVESYVVDVPDGNTEEDTRMFTDTVVKLNLQKLASVAEDSAAAPGSRRRD*

>TaPYL35

MDGGSSGVGADEIWRPWDEHTVLRPEEMEYVRQFHQHEPGANQCTSFIAKHIKAPLQTVWSLVRRFDEPQVFKPFVEKCVMQGNIEPGCVREVTIKSGLPGTWSTERLELLDDNEHILSVKFIDGDHPLKNYSSILTVHHEVIGGHPGALVIESFVVDIPEENTENEIFYLVGNFIKINHNLLADVSERRNRALN*

>TaPYL36

MMEAHMERALQEGVTEAERAALEGTVRAHHTFPGRVPGATCTSLVAQRVAAPVRAVWPIVRSFGNPQRYKHFVRTCALAAGDGASVGSVREVTVVSGLPASTSTERLEILDDDRHILSFSVVGGEHRLRNYRSVTSVTEFQPGPYCVVVESYVVDVPDGNTEEDTRMFTDTVVKLNLQKLASVAEETAAAPGSRRRD*

>TaPYL37

MDGGSSGVGADGIWRPWDEHTVLRPEEMEYVRRFHQHEPGANQCTSFIAKHIKAPLQTVWSVVRRFDKPQVYKRFVENCVMQGNIEPGCVREVTLKSGLPGKWSIERLELLDDNEHILSVKFIDGDHPLKNYSSILTVHHEVIDGHPGALVIESFVVDIPEENTKNEIFYLVGNFLKFNHKLLADVSEGRIDRRALN*

>TaPYL38

MMEAHMEQALREGVTEAERAALEGTVRAHHTFPGRAPGATCTSLVAQRVAAPVRAVWPIVRSFGNPQRYKHFVRTCALAAGDGASVGSVREVTVVSGLPASTSTERLEILDDDRHILSFSVVGGEHRLRNYRSVTSVTEFQPGPYCVVVESYVVDVPEGNTEEDTRMFTDTVVKLNLQKLASVAEESAAAPGSRRRD*

>PtPYRL1

MTDPAQQEPTTYTTHHVTIPPSLTQSEFDELNPLITEFHNYRIRPGQCSSLLAQRINAPNDLVWSLARRFDKPQTYKHFIKSCSVAPGFTMTVGSTRDVNVISGLPAATSTERLDILDDERQVTGFSIIGGEHRLKNYRSVTTVHGFEREGKIWTVVLESYVVDVPEGNTEEDTRLFADTVVKLNLQKLASVAEGLARDGDGK*

>PtPYRL10

MNGSDAYSATEAQYVRRHHKHEPRENQCTSALVKHIKAPAHLVWSLVRRFDQPQRYKPFVSRCVMNGELGIGSVREVNVKSGLPATTSTERLELLDDEEHILGVQIVGGDHRLKNYSSIMTVHPEFIDGRPGTLVIESFIVDVPDGNTKDETCYFVKALIRCNLKSLADVSERMAVQDRVEPVNQF*

>PtPYRL11

MNGNCNGRGGIGCVESEYIRRHHTHDDLADHQCSSALVKHIKAPVQLVWSLVRRFDQPQKYKPFISRCVVLGNLEIGSLREVDVRSGLPATTSTERLELLDDDEHILSIRIVGGDHRLKNYSSIISLHPEIIDGRPGTLVIESFVVDVPDGNTKDETCYFVEALIKCNLKSLADVSEHLAVQDRTEPIDCM*

>PtPYRL12

MVTNDYVTIASGKMEDEFIKRHHKHDVKEHQCSSSLVKHIKAPVPLVWSLVRRFDQPQKYKPFVSRCVVQGDLQIGSVREVNVKSGLPATTSTERLELLDDEEHIFSMKIVGGDHRLKNYLSTVTVHPEVIDGRPGTLVIESFIVDVPDGNTKDETCYFVEALIKCNLKSLADVSERLAVQDRTEPIDRM*

>PtPYRL13

MNENSNGRGGIGSVESEYIRRHHKHGDLADHQCSSALVKHIKAPVHLVWSLVRRFDQPQKYKPFISRCVVLGNLEIGSLREVDVRSGLPATTSTERLELLDDDEHIFSIRIVGGDHRLKNYSSVISLHPEIIDGRPGTLVIESFVVDVPDGNTKDETCYFVEALIKCNLKSLADVSESHAVQDRTEPIECM*

>PtPYRL14

MVTNDYVTIANGMMEDEFIKRHHKHDVKEHQCSSSLVKHIKAPVPLVWSLVRRFDQPQKYKPFVSRCIAQGDLQIGSVREVNVKSGLPATTSTERLELLDDDEHIFGMKIVGGDHRLKNYSSIVTVHPKVIDGRPGTLVIESFVVDVPDGNTKDETCYFVEALIKCNLKSLADVSERLAVQGRTEPIDRT*

>PtPYRL2

MSQKSKDMTDPEQQESIASTTHHITIPSGLTQSESEELAPLITEFHTYRISAGQCSSLLAQLISAPNDTVWSIVRRFDKPQTYKHFIKSCSVGPGFTMTVGSTRDVNVISGLPAATSTERLDILDDEQQLTGFSIIGGEHRLRNYRSVTTVHGFEREGKIRTVVLESYVVDVPEGNTEEEARLFADTVVKLNLQKLASVAESLVRDGDGK*

>PtPYRL3

MDTNQAPPPQGLTQEEYMELKPLIDTYHKFEPAPNTCTSLITQRIDAPAHVVWPFVRRFDNPQKYKHFIKSCNMSAGDGGVGSVREVAVVSGLPASTSIERLEILDDENHILSFRVVGGEHRLNNYKSVTSVNEFNKEGKVYAIVLESYIVDIPGGNTGEDTKMFVDTVVKLNLQKLAVVAIASLHGHE*

>PtPYRL4

MDANHAPPVPQGLTQEEYVELKPLIDTYHKFGAAVPNTCTSLITQRIDAPAHVVWPFVRRFDNPQKYKHFIKSCKMSAGDGGVGSIREVTVVSGIPASTSTERLEILDDENHILSFRVVGGEHRLNNYKSVTSVNEFNKEDKVYTIVLESYIVDIPDGNTVEDTEMFVDTVVKLNLQKLAVVANTALHGHE*

>PtPYRL5

MPASLQLQRAAIPTTTTTLACHKQSQTTVNTWRVPLTWDAPVPDYVSCHHTRLVGPKQCCSVVVKTINAPVSTVWSVVRRFDNPQAYKHFVKSCHVIDGDGNVGSLREVHVVSGLPAASSTERLEILDDEQHVLSFSVVGGVHRLNNYRSVTTLHASPNGNGTVVVESYVVDVPAGNTKEDTCSFIETIVRCNLQSLAQIAEKMARNAQISTSS*

>PtPYRL6

MPANPPKSSLLIHRINNTTITNTTLNTTNTTTSTSCQKRWSPLTCATIPVPETVSRYHAHAVGPNQCCSAVVQQIAAPVSTVWSVVRHFDNPQAYKHFVKSCHVILGDGDVGTLREVHVISGLPAAKSTERLEILDHERHVISFSVVGGDHRLANYRSVTTLHASPTGNGTVVVESYVVDIPPGNTKEDTCVFVDTIVRCNLQSLAQIAENKNRRNNKSSS*

>PtPYRL7

MYLSLTDHRKKKAKRFKMPASLQLQRAAATSTTTTRTGHKQSQTTVNTWGVPLPWDTPVPDYVSCHHTRIPGPNQCCSVVVQTINAPVATVWSVVRRFDNPQAYKHFLKSCHVIDGDGKVGSLREVHVVSGLPAASSTERLEILDDEQHILSFSVVGGVHRLNNYRSVTTLHASPNGNGTVVVESYVVDVPTGNTKEDTCSFLDTIVRCNLQSLAQIAGKKARNNQISITS*

>PtPYRL8

MPANPPRSSLLIHRINNTTSNTTLNTTNTTTATSCQKRWSPLPCDATPVPETVSRYHTHAVGPNQCCSAVVQQIAAPISTVWSVVRRFDNPQAYKHFVKSCHVILGDGDVGTLREIHVISGLPAAHSTERLEILDDERHVISFSVVGGDHRLANYKSVTTLHSSPSGNGTVVMESYAVDIPPGNTKEDTCVFVDTIVRCNLQSLAQIAENSNRRNNKSSSA*

>PtPYRL9

MNGGDAYSAAEVQYIRRHHQHEPAENQCTSALVKHIKAPAHLVWSLVRRFDQPQRYKPFVSRCVMNGELGIGSVREVNVKSGLPATTSTERLELLDDEEHILGVKIVGGDHRLKNYSSIMTVHPEIIDGRPGTLVIESFIVDVPDGNTKDETCYFVKALIRCNLKSLADVSERMAVQDLVEPINQF*
