# Supplementary material for: Identification of Malus sieversii ABA receptor PYL8 interacting proteome using Y2H-seq
Source: For Res (Fayettev). 2025 Jun 30;5:e012. doi: 10.48130/forres-0025-0012 (PMC12441796; doi:10.48130/forres-0025-0012)
Supplement: Supplementary file 1 — Supplementary data to this article can be found online. [file FR-2025-5-0012-Supplementary.zip › 10.48130_forres-0025-0012-Suppl-TableS1.pdf]

Supplemental Table S1. Primer name and sequences used in this experiment

Primer names	Sequence (5′-3′)	Application
CDS III/3' PCR Primer	AAGCAGTGGTATCAACGCAGAGTGGCCATTATGGCCGGG	Reverse transcriptase
SMART IV Oligonucleotide	ATTCTAGAGGCCGAGGCGGCCGACATGTTTTTTTTTTTTTTTTTTTTTTTTTTTTTTVN	
P1-F	TACGATGTTCCAGATTACGCTGGATCCAAGCAGTGGTATCAACGCAGAGTGG	ds cDNA amplification
P2-F	TACGATGTTCCAGATTACGCTGGATCCAAAGCAGTGGTATCAACGCAGAGTGG	
P3-F	TACGATGTTCCAGATTACGCTGGATCCAAAAGCAGTGGTATCAACGCAGAGTGG	
P4-R	GGTATCGATAAGCTTGATATCGAATTCCTAGAGGCCGAGGCGGCCGACATG	
Prime M1	AAGCAGTGGTATCAACGCAGAGT	Homogenization
pPR3-N-F	CGGTAAAACCGGAACATTGGA	Colony PCR
pPR3-N-R	ACTTCAGGTTGTCTAACTCCT	
MsPYL8-F	ATCCTACGTCGTCGATCTGC	Real-time fluorescence PCR
MsPYL8-R	CGCAGCTTCTGGAGATTGA	
βactin-F	CAGCATCACTACCATCTGCAAC	Internal reference gene
βactin-R	CCGCCATCTTCTACTTCCTGTTT	

Notes: V: A, C or G; N: A, T, C or G; The red part is the pPR3-N vector homologous arm. P1-4-F differ by one nucleotide in length at the back of the TACGATGTTCCAGATTACGCTGGATCCAA to permit expression of ORFs in all the 3 possible reading frames.
