# Supplementary material for: Identification of Malus sieversii ABA receptor PYL8 interacting proteome using Y2H-seq
Source: For Res (Fayettev). 2025 Jun 30;5:e012. doi: 10.48130/forres-0025-0012 (PMC12441796; doi:10.48130/forres-0025-0012)
Supplement: Supplementary file 1 — Supplementary data to this article can be found online. [file FR-2025-5-0012-Supplementary.zip › 10.48130_forres-0025-0012-Suppl-TableS2.pdf]

Supplemental Table S2. Yeast transformation reaction

Reaction	pBT3-SUC plasmid	pPR3-N plasmid	Conversion plate	Note
1	pBT3-SUC-PYL8(PYR1)	pPR3-N-LOC103406509	SD-TL	Experimental group
2	pBT3-SUC	pPR3-N-LOC103406509	SD-TL	Control group
3	pBT3-SUC-PYL8(PYR1)	pPR3-N	SD-TL	Control group
4	pBT3-SUC-LOC103406509	pPR3-N-LOC103445100	SD-TL	Experimental group
5	pBT3-SUC	pPR3-N-LOC103445100	SD-TL	Control group
6	pBT3-SUC-LOC103406509	pPR3-N	SD-TL	Control group
7	pBT3-SUC-LOC103445100	pPR3-N-LOC103430245	SD-TL	Experimental group
8	pBT3-SUC	pPR3-N-LOC103430245	SD-TL	Control group
9	pBT3-SUC-LOC103445100	pPR3-N	SD-TL	Control group
10	pBT3-SUC-YF12	pPR3-N-LOC101266657	SD-TL	Positive control
