# Supplementary material for: Identification of Malus sieversii ABA receptor PYL8 interacting proteome using Y2H-seq
Source: For Res (Fayettev). 2025 Jun 30;5:e012. doi: 10.48130/forres-0025-0012 (PMC12441796; doi:10.48130/forres-0025-0012)
Supplement: Supplementary file 1 — Supplementary data to this article can be found online. [file FR-2025-5-0012-Supplementary.zip › 10.48130_forres-0025-0012-Suppl-TableS3.pdf]

Supplemental Table S3. Secondary structure of PYL protein in *Malus sieversii*

Protein name	Alpha helix	Extended strand	Beta turn	Random coil
MsPYL1	41.30%	14.67%	3.80%	40.22%
MsPYL2	35.86%	14.65%	5.56%	43.94%
MsPYL3	38.69%	15.48%	1.79%	44.05%
MsPYL4	22.41%	20.69%	4.31%	52.59%
MsPYL5	43.12%	14.37%	1.88%	40.62%
MsPYL6	35.20%	13.20%	1.20%	50.40%
MsPYL7	41.08%	14.59%	0.00%	44.32%
MsPYL8	35.92%	15.05%	3.88%	45.15%
MsPYL9	37.31%	12.94%	2.99%	46.77%
MsPYL10	37.23%	14.32%	3.10%	45.35%
MsPYL11	38.14%	12.37%	2.58%	46.91%
MsPYL12	37.35%	15.66%	1.81%	45.18%
MsPYL13	39.11%	14.36%	2.97%	43.56%
MsPYL14	20.75%	17.35%	4.42%	57.48%
