# Supplementary material for: Identification of Malus sieversii ABA receptor PYL8 interacting proteome using Y2H-seq
Source: For Res (Fayettev). 2025 Jun 30;5:e012. doi: 10.48130/forres-0025-0012 (PMC12441796; doi:10.48130/forres-0025-0012)
Supplement: Supplementary file 1 — Supplementary data to this article can be found online. [file FR-2025-5-0012-Supplementary.zip › 10.48130_forres-0025-0012-Suppl-TableS4.pdf]

Supplemental Table S4. FPKM values of *MsPYL* gene in six tissues

Gene	Flower	Fruit	Seed	Root	Stem	Leaf
MsPYL1	8.525903	8.713096	8.798732	8.300605	8.609985	8.386056
MsPYL2	8.264071	8.325827	6.891914	8.092755	7.992544	7.704177
MsPYL3	11.57418	11.12366	12.82776	11.32366	11.66815	10.35289
MsPYL4	8.362789	8.553135	7.572925	8.127077	8.159575	7.920278
MsPYL5	8.75917	8.868615	7.841626	8.32761	8.106504	8.598836
MsPYL6	7.96471	8.112283	7.538706	7.973615	8.094505	8.078092
MsPYL7	10.79385	11.34771	10.06891	9.590906	9.539911	10.74797
MsPYL8	11.8625	12.65257	10.56492	11.11371	11.02414	12.43204
MsPYL9	11.41499	10.87966	11.70866	10.59466	11.46575	8.61545
MsPYL10	8.006274	7.893877	7.158827	8.232497	8.078642	7.837832
MsPYL11	8.950029	9.202264	7.694762	8.158917	7.993528	8.216937
MsPYL12	8.218592	8.513792	7.537181	10.16001	8.363477	8.06441
MsPYL13	10.76559	10.83725	11.4841	10.89504	10.73371	10.92463
MsPYL14	8.377221	8.168909	7.589923	8.028602	8.458852	8.004485
