# Supplementary figures and images for: Identification of Malus sieversii ABA receptor PYL8 interacting proteome using Y2H-seq
Source: For Res (Fayettev). 2025 Jun 30;5:e012. doi: 10.48130/forres-0025-0012 (PMC12441796; doi:10.48130/forres-0025-0012)

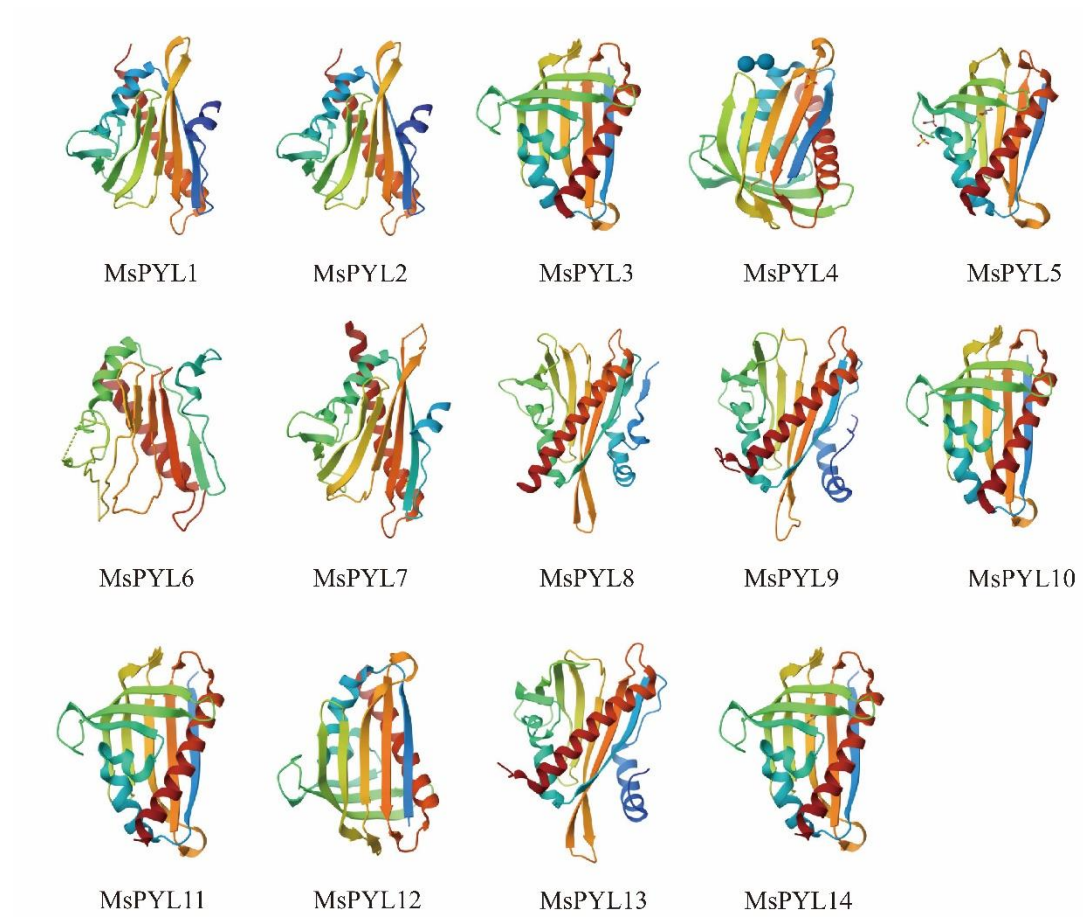

**Supplemental Fig.S1** The tertiary structure of MsPYL protein in *Malus sieversii*

Supplement: Supplementary file 1 — Supplementary data to this article can be found online. [file FR-2025-5-0012-Supplementary.zip › 10.48130_forres-0025-0012-Suppl-FigureS1.pdf]

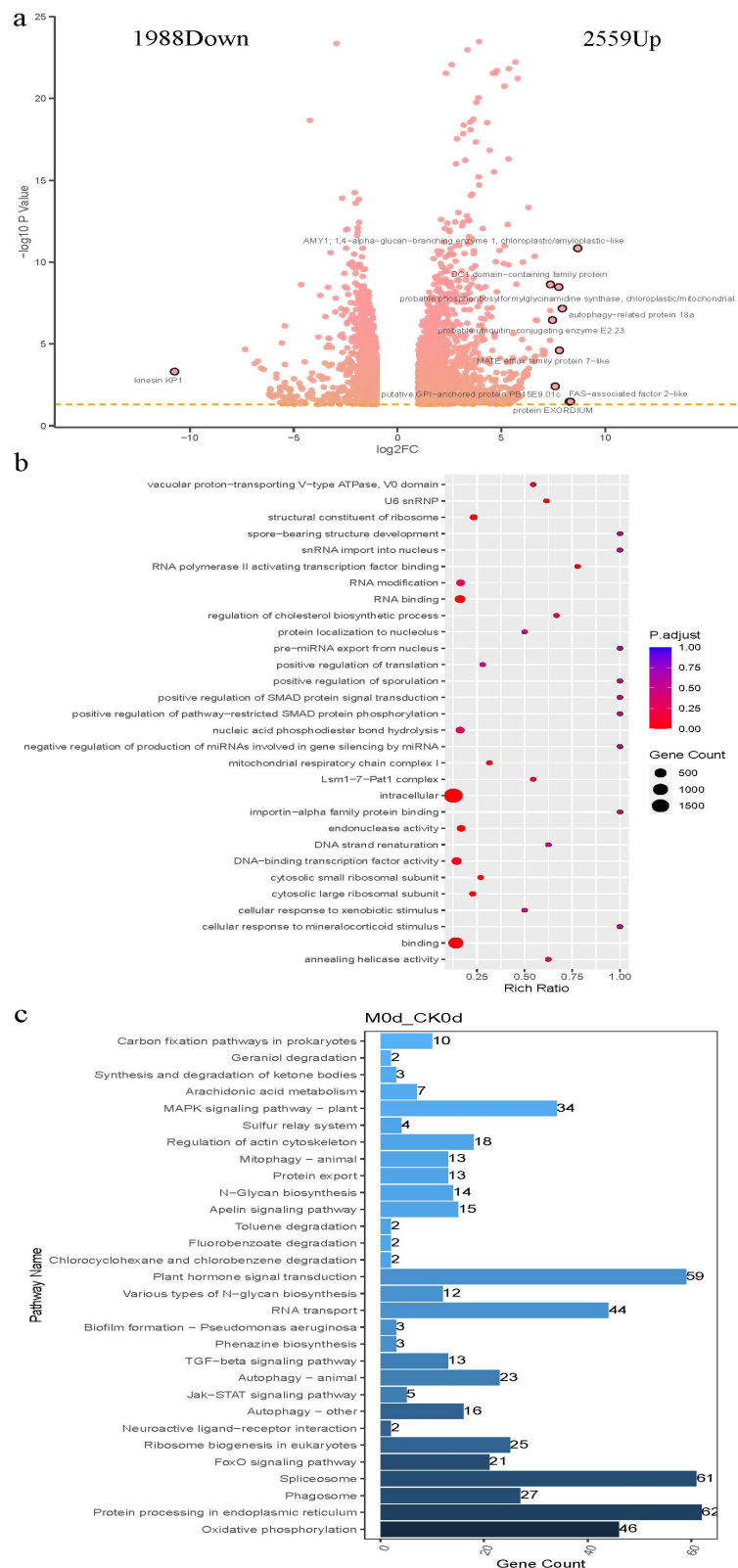

Supplement: Supplementary file 1 — Supplementary data to this article can be found online. [file FR-2025-5-0012-Supplementary.zip › 10.48130_forres-0025-0012-Suppl-FigureS4.pdf]
